# Reporting bias in the literature on the associations of health-related behaviors and statins with cardiovascular disease and all-cause mortality

**DOI:** 10.1371/journal.pbio.2005761

**Published:** 2018-06-18

**Authors:** Leandro Fórnias Machado de Rezende, Juan Pablo Rey-López, Thiago Hérick de Sá, Nicholas Chartres, Alice Fabbri, Lauren Powell, Emmanuel Stamatakis, Lisa Bero

**Affiliations:** 1 Departamento de Medicina Preventiva, Faculdade de Medicina FMUSP, Universidade de São Paulo, São Paulo, São Paulo, Brasil; 2 Prevention Research Collaboration, School of Public Health, The University of Sydney, Sydney, Australia; 3 Núcleo de Pesquisas Epidemiológicas em Nutrição e Saúde, Universidade de São Paulo, São Paulo, São Paulo, Brasil; 4 Charles Perkins Centre, Faculty of Pharmacy, The University of Sydney, Sydney, New South Wales, Australia; 5 Charles Perkins Centre, Epidemiology Unit, The University of Sydney, Sydney, Australia; University of Edinburgh, United Kingdom of Great Britain and Northern Ireland

## Abstract

Reporting bias in the literature occurs when there is selective revealing or suppression of results, influenced by the direction of findings. We assessed the risk of reporting bias in the epidemiological literature on health-related behavior (tobacco, alcohol, diet, physical activity, and sedentary behavior) and cardiovascular disease mortality and all-cause mortality and provided a comparative assessment of reporting bias between health-related behavior and statin (in primary prevention) meta-analyses. We searched Medline, Embase, Cochrane Methodology Register Database, and Web of Science for systematic reviews synthesizing the associations of health-related behavior and statins with cardiovascular disease mortality and all-cause mortality published between 2010 and 2016. Risk of bias in systematic reviews was assessed using the ROBIS tool. Reporting bias in the literature was evaluated via small-study effect and excess significance tests. We included 49 systematic reviews in our study. The majority of these reviews exhibited a high overall risk of bias, with a higher extent in health-related behavior reviews, relative to statins. We reperformed 111 meta-analyses conducted across these reviews, of which 65% had statistically significant results (*P* < 0.05). Around 22% of health-related behavior meta-analyses showed small-study effect, as compared to none of statin meta-analyses. Physical activity and the smoking research areas had more than 40% of meta-analyses with small-study effect. We found evidence of excess significance in 26% of health-related behavior meta-analyses, as compared to none of statin meta-analyses. Half of the meta-analyses from physical activity, 26% from diet, 18% from sedentary behavior, 14% for smoking, and 12% from alcohol showed evidence of excess significance bias. These biases may be distorting the body of evidence available by providing inaccurate estimates of preventive effects on cardiovascular and all-cause mortality.

## Author summary

In the scientific literature, reporting bias occurs when communication and publication of results are influenced by the direction of findings. Reporting bias can distort scientific evidence and may misguide subsequent clinical and public health efforts. Our study provided an assessment of the degree of reporting bias in the literature on health-related behavior (smoking, alcohol, diet, physical activity, and sedentary behavior) and statins and their association with cardiovascular disease and mortality. We analyzed recently published systematic reviews. Most of the systematic reviews (90%) had a high risk of bias related to study eligibility criteria, identification and selection of studies, data collection and study appraisal, and synthesis and findings. We found evidence of reporting bias in about one-fifth of health-related behavior meta-analyses but none of the statin-related meta-analyses. Readers should be aware of the extent of reporting bias in these research areas when interpreting meta-analytical results.

## Introduction

The literature on the association between behavioral risk factors (e.g., smoking, alcohol, physical inactivity, and unhealthy diet) and cardiovascular diseases—the single largest cause of death globally [1]—has grown exponentially in the last decades [2–39]. Observational epidemiological studies are the dominant design assessing these associations, since clinical trials cannot always be ethically or logistically conducted [40]. Systematic review methods are used to synthesize and evaluate this growing body of evidence. It is important to evaluate the methodological risks of bias in systematic reviews [41], as well as the impact that reporting bias can have on the findings of reviews [42, 43].

Reporting bias is one of the most common biases identified in the literature. It includes selective publication of studies or outcomes of studies [44, 45] based on factors other than the study quality, such as nominally statistically significant results (*P* < 0.05) [46, 47] or authors’ “pedigree” [44, 45, 48]. These practices threaten the completeness and validity of scientific evidence [46] by distorting the estimates of causal effects of interventions or exposures on diseases [49]. The extent of reporting bias could differ between bodies of evidence consisting of randomized trials, such as drug studies, compared to observational studies, such as studies of health behavior. Different levels of reporting bias in the literature on health behavior may lead to inaccurate estimates of preventive effects on cardiovascular and all-cause mortality and therefore offer incorrect guidance for policymaking.

To gain a better understanding of the potential reporting bias in the literature on health-related behavior and cardiovascular disease mortality and all-cause mortality, we examined reporting and other risks of bias in a sample of systematic reviews published between 2010 to 2016. Our analysis also provided a comparative assessment of the reporting bias between health-related behavior and statins used in primary prevention.

## Results

**Fig 1:**
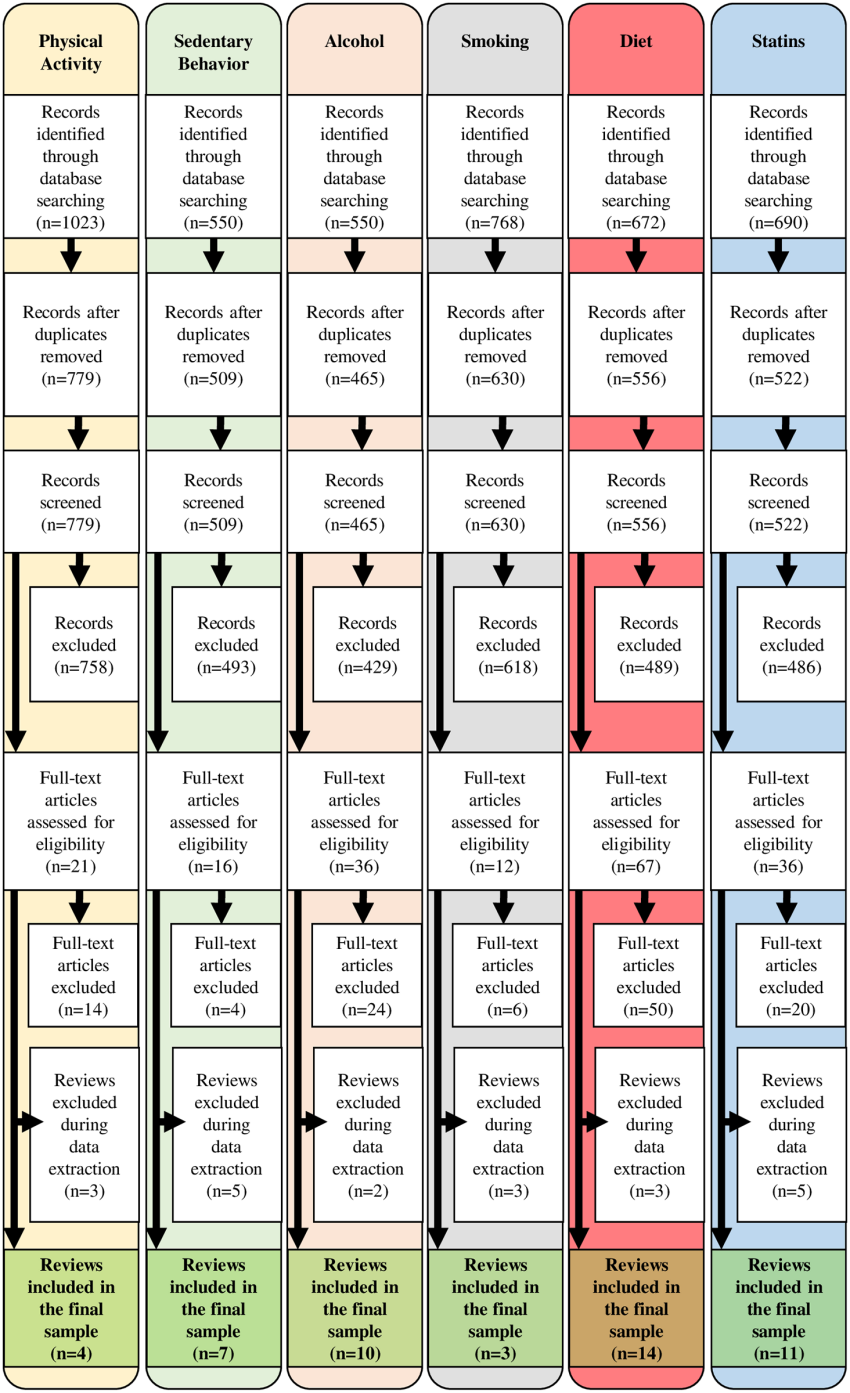
Flowchart for systematic review selection by research area.

Of the 5,511 records identified while searching the databases, we selected 49 systematic reviews. All research areas (tobacco, alcohol, diet, physical activity, sedentary behavior, and statins) presented fewer than 20 eligible systematic reviews; therefore, we included all the systematic reviews within each area that met our inclusion criteria (Fig 1). Lists of excluded reviews and reasons for exclusions are described in S1 and S2 Tables. Studies were excluded most frequently for not including one of the exposures (28%) or outcomes (29%) of interest and for utilizing clinical samples (16%).

Most of the included systematic reviews (*n* = 35, 71.4%) analyzed only one outcome eligible for our study (cardiovascular disease mortality and all-cause mortality), whereas 9 (18.4%), 4 (8.2%), and 1 (2.0%) analyzed two, three, and four outcomes, respectively. All-cause mortality (69%), cardiovascular disease mortality (29%), and stroke mortality (14%) were the most frequent outcomes investigated (Table 1).

**Table 1:** Risk of bias in systematic reviews of the associations of health-related behavior and statins with cardiovascular and all-cause mortality—ROBIS assessment.

First author, year (reference)	Exposures	Outcomes (mortality)		ROBIS Assessment		
			1. Study eligibility criteria	2. Identification and selection of studies	3. Data collection and study appraisal	4. Synthesis and findings
**Physical activity**						
Kelly, 2014 [2]	Walking; Cycling	ACM	low risk	high risk	high risk	high risk
Samitz, 2011 [3]	Total leisure time; Exercise; Walking; Commuting; Daily activities	ACM	low risk	low risk	high risk	high risk
Woodcock, 2011 [4]	Nonvigorous; Walking	ACM	high risk	high risk	unclear risk	low risk
Hupin, 2015 [5]	Low-dose physical activity	ACM	low risk	high risk	unclear risk	low risk
**Sedentary behavior**						
Biswas, 2015 [6]	Sedentary time	CVD, ACM	low risk	high risk	low risk	high risk
Chau, 2013 [7]	Sedentary time	ACM	high risk	high risk	low risk	high risk
Grontved, 2011 [8]	Television viewing	ACM	low risk	high risk	high risk	high risk
Wilmot, 2012 [9]	Sedentary time	CVD, ACM	high risk	high risk	low risk	high risk
Ford, 2012 [10]	Sitting time; Screen time	CVD	high risk	high risk	high risk	high risk
Pandey, 2016 [11]	Sedentary time	CVD	high risk	high risk	unclear risk	unclear risk
Sun, 2015 [12]	Television viewing	ACM	high risk	unclear risk	high risk	high risk
**Alcohol**						
Costanzo, 2011 [13]	Wine, Beer, Spirits	CHD, CVD, ACM	high risk	high risk	unclear risk	high risk
Jayasekara, 2014 [14]	Alcohol intake	ACM	low risk	high risk	high risk	high risk
Roerecke, 2011 [15]	Alcohol intake	IHD	low risk	high risk	high risk	low risk
Roerecke, 2014 [16]	Heavy drinking	IHD	low risk	high risk	high risk	high risk
Ronksley, 2011 [17]	Alcohol intake	CVD, CHD, Stroke, ACM	low risk	low risk	high risk	low risk
Park, 2015 [18]	Moderate alcohol intake	ACM	high risk	high risk	high risk	high risk
Stockwell, 2016 [19]	Low alcohol intake	ACM	low risk	high risk	unclear risk	low risk
Zheng, 2015 [20]	Alcohol intake	ACM, Cardiac death	high risk	low risk	low risk	low risk
Roerecke, 2010 [21]	Alcohol intake	IHD	high risk	high risk	high risk	high risk
Roerecke, 2014 [22]	Alcohol intake	IHD	low risk	high risk	high risk	high risk
**Smoking**						
Gellert, 2012 [23]	Current smoker; Former smoker	ACM	low risk	unclear risk	unclear risk	unclear risk
Lv, 2015 [24]	Secondhand smoking	ACM, CVD	low risk	high risk	unclear risk	high risk
Sinha, 2016 [25]	Secondhand smoking	ACM, IHD, Stroke	low risk	high risk	high risk	high risk
**Diet**						
Farvid, 2014 [26]	Dietary linoleic acid	ACM	low risk	high risk	high risk	high risk
Graudal, 2014 [27]	Sodium	ACM	low risk	high risk	high risk	high risk
Hu, 2014 [28]	Fruits and vegetables	Stroke	low risk	high risk	high risk	high risk
Li, 2012 [29]	Salt intake	Stroke	high risk	high risk	high risk	high risk
Musa-Veloso, 2011 [30]	Long-chain n-3 fatty acid	Sudden cardiac, Coronary event	high risk	high risk	high risk	high risk
Pan, 2012 [31]	α-linolenic acid	CVD	high risk	high risk	low risk	high risk
Poggio, 2015 [32]	Sodium	CVD	low risk	high risk	high risk	low risk
Schwingshackl, 2014 [33]	MUFA; MUFA:SFA ratio, olive oil	ACM, CVD	low risk	low risk	high risk	high risk
Wang, 2014 [34]	Fruits and vegetables	ACM, CVD	low risk	high risk	low risk	high risk
Chen, 2016 [35]	Long-chain n-3 polyunsaturated; EPA; DHA	ACM	low risk	low risk	low risk	low risk
Cheng, 2015 [36]	Long chain n-3 PUFA intake	Stroke	low risk	high risk	low risk	high risk
Cheng, 2016 [37]	Dietary saturated fat	Stroke	low risk	high risk	low risk	high risk
De Souza, 2015 [38]	Saturated fat; total trans fat; industrial trans fat; Ruminants’ trans fat	CHD	low risk	high risk	high risk	low risk
Narain, 2016 [39]	Artificially sweetened beverage	ACM	low risk	low risk	high risk	low risk
**Statins**						
Bukkapatnam, 2010 [50]	Statin	ACM	high risk	high risk	high risk	high risk
Kizer, 2010 [51]	Statin	ACM	low risk	high risk	high risk	high risk
Kostis, 2012 [52]	Statin	ACM	high risk	high risk	high risk	low risk
Lv, 2014 [53]	Statin	ACM, CVD	high risk	high risk	high risk	low risk
Ray, 2010 [54]	Statin	ACM	high risk	high risk	high risk	high risk
Savarese, 2013 [55]	Statin	CVD, ACM	low risk	high risk	low risk	low risk
Taylor, 2011 [56]	Statin	CHD, CVD, ACM	low risk	low risk	low risk	low risk
Tonelli, 2011 [57]	Low-dose statin; High-dose statin	ACM	low risk	high risk	low risk	high risk
Chou, 2016 [58]	Statin	ACM	low risk	high risk	low risk	low risk
Preiss, 2015 [59]	Statin	Heart failure	low risk	high risk	high risk	low risk
Teng, 2015 [60]	Statin	Stroke, MI, ACM	low risk	high risk	low risk	low risk

low risk = low risk of bias; high risk = high risk of bias; unclear risk = unclear risk of bias

**Abbreviations**: ACM, all-cause mortality; CHD, coronary heart disease; CVD, cardiovascular disease; DHA, docosahexaenoic acid; EPA, eicosapentaenoic acid; IHD, ischemic heart disease; MI, myocardial infarction; MUFA, monounsaturated fatty acid; SFA, saturated fatty acid; PUFA, polyunsaturated fatty acid

### Risk of bias in systematic reviews—ROBIS results

**Fig 2:**
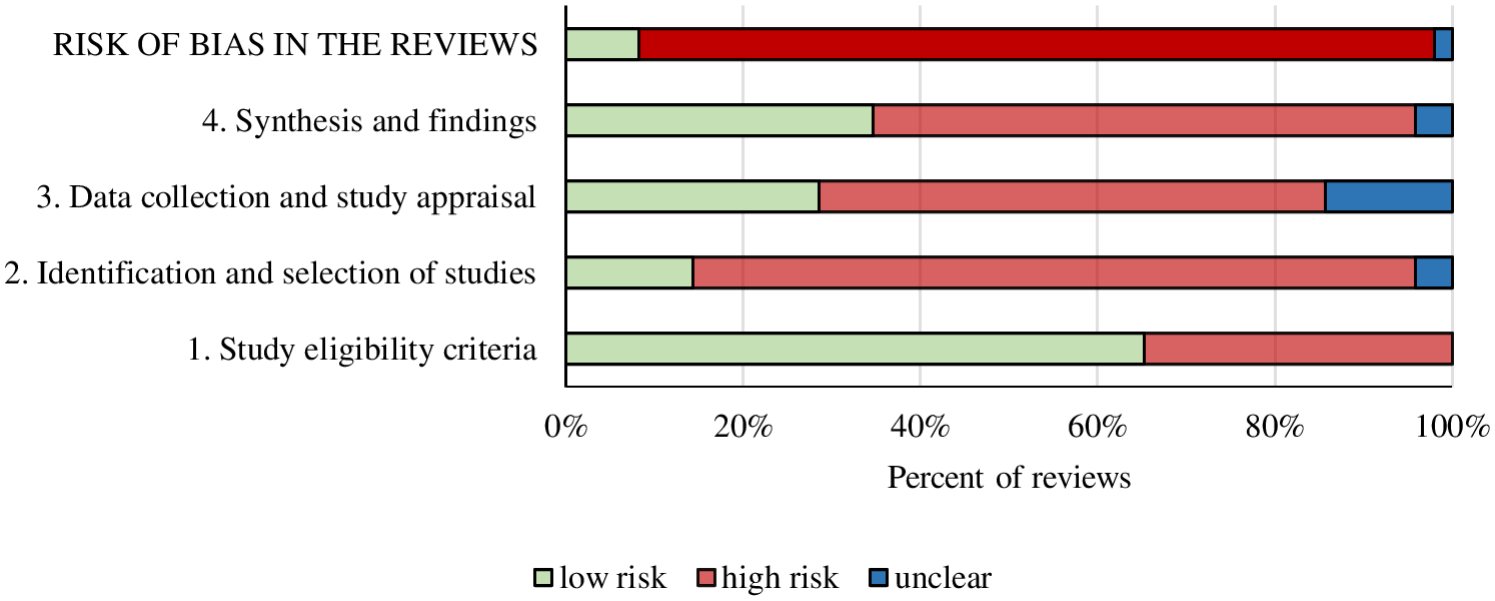
Risk of bias in systematic reviews of the associations of health behavior and statins with cardiovascular disease mortality and all-cause mortality—ROBIS results. Underlying data can be found in S1 Data.

The majority of the systematic reviews exhibited a high overall risk of bias (*n* = 44, 90%) (Fig 2). Among the four ROBIS domains, domain 1 (study eligibility criteria) presented the best scores, with 32 (65%) out of 49 reviews showing a low risk of bias. In domain 2 (identification and selection of studies), 2 (4%) reviews were scored as unclear, 40 (82%) showed a high risk, and 7 (14%) a low risk of bias. Whereas, in domain 3 (data collection and study appraisal), 7 (14%) reviews were scored as unclear, 28 (57%) scored with high risk, and 14 (29%) with low risk of bias. Finally, in domain 4 (synthesis and findings), 2 (4%) review was scored as unclear, 30 (61%) with high risk, and 17 (35%) with low risk of bias (Fig 2 and S3 Table).

**Fig 3:**
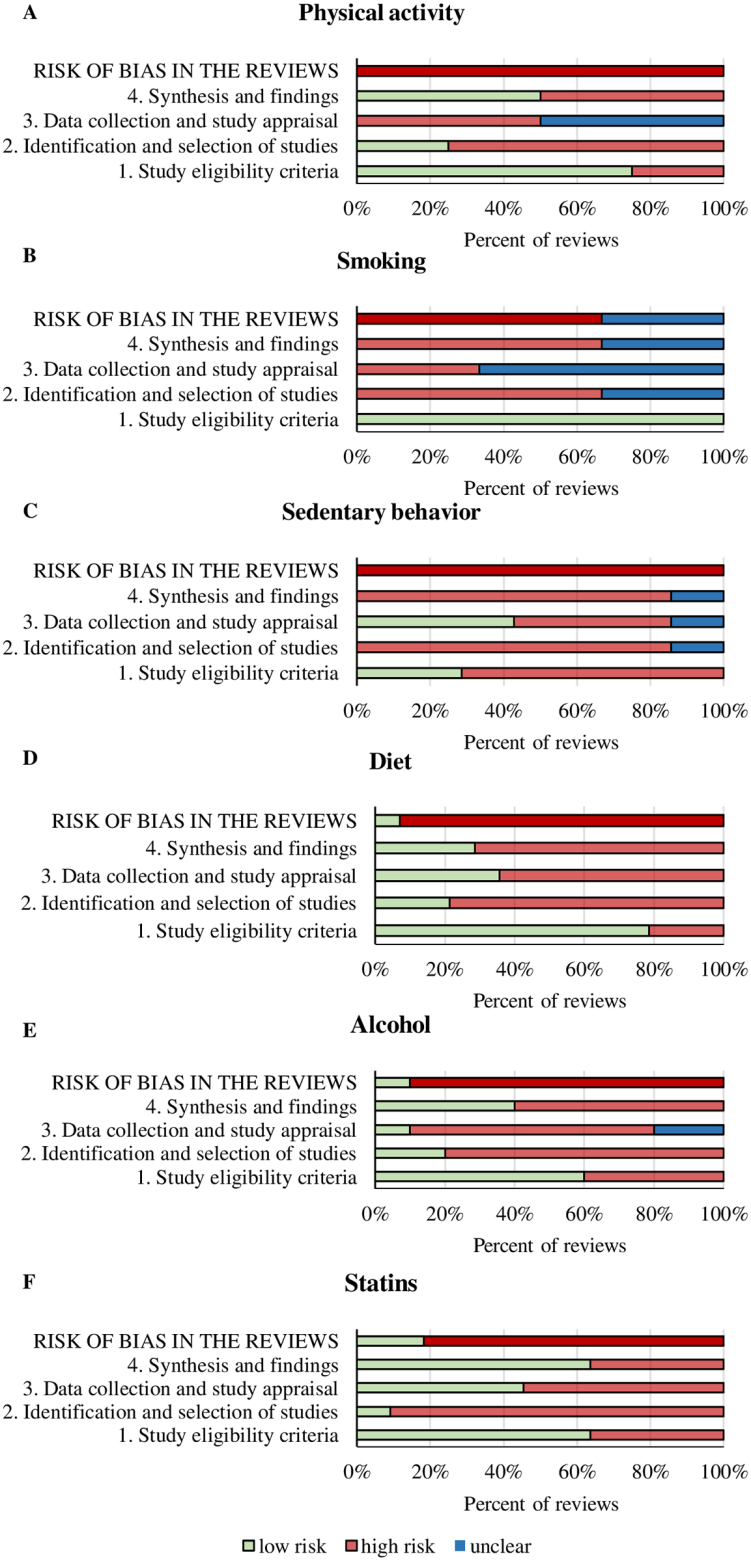
Risk of bias in systematic reviews of the associations of health behavior and statins with cardiovascular disease mortality and all-cause mortality, by research area—ROBIS results. Risk of bias in systematic reviews of the associations of physical activity (A), smoking (B), sedentary behavior (C), diet (D), alcohol (E), and statins (F) with cardiovascular disease mortality and all-cause mortality. Underlying data can be found in S1 Data.

Comparing risk of bias in the reviews across research areas, sedentary behavior performed worst in domain 1 (study eligibility criteria; 70% of reviews were regarded as having high risk of bias). All research areas performed poorly in domain 2 (identification and selection of studies), with high risk of bias ranging from 70% in smoking reviews to 90% in both sedentary behavior and statin reviews. Alcohol (70%) and diet (60%) reviews presented high risk of bias in domain 3 (data collection and study appraisal). Sedentary behavior (90%), smoking (70%), and diet (70%) reviews presented high risk of bias in domain 4 (synthesis and findings). Overall, statin reviews presented the best scores in the ROBIS assessment compared to other research areas. Among statin reviews, a low risk of bias was identified in 60% in domain 1, 10% in domain 2, 50% in domain 3, and 60% in domain 4 (Table 1 and Fig 3).

### Risk of reporting bias in the body of evidence

We identified 111 meta-analyses (exposure–outcomes associations) that were performed across the 49 included reviews. On average, each meta-analysis synthesized results from 9 primary studies (ranging from 2 to 81), including 331,688 participants (ranging from 595 to 3,674,042) and 19,012 deaths (ranging from 33 to 320,252) (Table 2 and S4 Table). Of the 111 meta-analyses, 72 (65%) showed a nominally statistically significant result at *P* < 0.05.

**Table 2:** Relative and absolute frequency of meta-analyses with nominally statistically significant results, small-study effect, and excess significance, by research area.

Research area	Total number of meta-analyses	Meta-analyses with *P* < 0.05^#^		Small-study effect			Excess significance				
	*N*	*n*	Percent	N*	*n*	Percent	N**	O > E			
								*n*	Percent	*n* with *P* < 0.05	Percent
Physical activity	12	12	100	12	5	42	12	8	67	6	50
Sedentary behavior	12	11	92	11	1	9	11	7	64	2	18
Alcohol	24	9	38	18	3	17	17	10	59	2	12
Smoking	7	7	100	7	3	43	7	3	43	1	14
Diet	36	24	67	31	5	16	31	16	52	8	26
Statin	20	9	45	17	0	0	17	9	53	0	0
Overall	111	72	65	96	17	18	95	53	56	19	20

**Abbreviations**: E, number of expected primary studies with *P* < 0.05 results; O, number of observed primary studies with *P* < 0.05 results

*N: Number of meta-analyses with enough primary studies to perform the small-study effect test (≥3)

**N: Number of meta-analyses with enough primary studies (≥3) and available data to perform the excess significance test

^#^ *P*-value of the summary random effects estimate

Nominally statistically significant results (*P* < 0.05) were found in 92% of the meta-analyses from sedentary behavior and 100% of the meta-analyses from physical activity and smoking. Alcohol and statin reviews had 38% and 45% of meta-analyses with *P* < 0.05 results, respectively (Table 2 and S4 Table).

#### Small-study effect

Indication of small-study effect was found in 17 (18%) of the 96 meta-analyses. Physical activity and the smoking research areas had more than 40% of meta-analyses with small-study effect. Sedentary behavior had less than 10% of meta-analyses with small-study effect (Table 2 and S4 Table). Overall, 17 (22%) out of 79 meta-analyses of health-related behavior presented small-study effect, as compared to zero-statin meta-analyses.

#### Excess significance

More than half (56%; 53/95) of the meta-analyses displayed a greater number of observed primary studies with *P* < 0.05 results (O) than the number number of expected primary studies with *P* < 0.05 results (E). Of those, 19 meta-analyses (20% of the total meta-analyses and 36% of the meta-analyses with O > E) showed evidence of excess significance bias (*P* < 0.10). Half of the meta-analyses from physical activity, 26% from diet, 18% from sedentary behavior, 14% from smoking, and 12% from alcohol showed evidence of excess significance (Table 2 and S4 Table). Overall, 24% of the meta-analyses of health-related behavior showed excess significance, as compared to zero-statin meta-analyses.

### Sensitivity analyses

We conducted a sensitivity analysis by restricting the sample in each research area to meta-analyses with ≥10 primary studies. In this subsample (*n* = 29), 86% of the meta-analyses showed statistically significant results at *P* < 0.05, as compared to 65% in the entire sample of meta-analyses. These results varied by research area, ranging from 60% in statin meta-analyses to 100% in physical activity, sedentary behavior, and smoking meta-analyses (Table 3).

**Table 3:** Sensitivity analysis: Relative and absolute frequency of meta-analyses with ≥10 primary studies showing nominally statistically significant results, small-study effect, and excess significance, by research area.

Research area	Total number of meta-analyses	Meta-analyses with *P* < 0.05^#^		Small-study effects			Excess significance				
	*N*	*n*	Percent	N*	*n*	Percent		O > E			
							N**	*n*	Percent	*n* with *P* < 0.05	Percent
Physical activity	5	5	100	5	4	80	5	2	40	2	40
Sedentary behavior	2	2	100	2	1	50	1	1	100	1	100
Alcohol	7	6	86	7	2	29	5	2	40	1	20
Smoking	6	6	100	6	2	33	6	3	50	1	17
Diet	4	3	75	4	0	0	4	3	75	2	50
Statin	5	3	60	5	0	0	5	4	80	0	0
Overall	29	25	86	29	9	31	26	15	58	7	27

**Abbreviations**: E, number of expected primary studies with *P* < 0.05 results; O, number of observed primary studies with *P* < 0.05 results

*N: Numbers of meta-analyses with enough primary studies to perform the small-study effect test (≥10 primary studies)

**N: Numbers of meta-analyses with enough primary studies (≥10) and available data to perform the excess significance test

^#^ *P*-value of the summary random effects estimate

Small-study effect was present in 31% of the meta-analyses. The proportions of meta-analyses in the sensitivity analysis with small-study effect were 80% for physical activity, 50% for sedentary behavior, 29% for alcohol, and 33% for smoking. Diet and statin meta-analyses had no evidence of small-study effect. Around 38% of the health-related behavior meta-analyses with ≥10 primary studies presented small-study effect, as compared to zero in statin meta-analyses (Table 3).

Excess significance was identified in 27% of the meta-analyses with ≥10 primary studies: 100% of the meta-analyses for sedentary behavior, 50% for diet, 40% for physical activity, 20% for alcohol, and 17% for smoking. Around 33% of the health-related meta-analyses with ≥10 primary studies showed evidence of excess significance, as compared to zero in statin meta-analyses (Table 3).

Overall, after excluding small individual studies (with <200 deaths) from meta-analyses, results from small-study effect and excess significance tests did not change (S5 Table).

## Discussion

This study aimed to assess the extent of reporting bias among recent meta-analyses that examined the associations of health behavior and statins with cardiovascular and all-cause mortality. We found evidence of reporting bias across all health-related behavior areas. The degree of reporting bias varied by the method used to assess it. Reporting bias was present in 20% (according to excess significance test) or 18% (according to small-study effect test) of all meta-analyses included (health behavior and statins). Evidence of reporting bias was found in between a quarter and one-fifth of health-related behavior meta-analyses (22% small-study effect and 24% excess significance) but in none of the statin meta-analyses (0%).

In lifestyle epidemiology, the interpretation of evidence for researchers and policymakers is challenging for several reasons [61]. As observational studies are the dominant designs in this area, spurious associations can arise due to confounding or several sources of bias. The impact of such biases on statistical findings and interpretation of findings has been poorly reported and discussed [62]. Therefore, meta-analytical synthesis of the evidence in lifestyle health behavior epidemiology may provide precise but spurious results [63].

Reporting bias is a major threat to the validity of the relevant body of evidence. Our results suggest that around 20% of the meta-analyses on health-related behavior and cardiovascular disease mortality and all-cause mortality may be susceptible to reporting biases. The existence of reporting bias in the literature has several explanations. Failure to submit manuscripts of analyses that did not produce statistically significant results (“the file-drawer problem” [46]) and the low likelihood of publication of small studies (regardless of statistical significance) [44] are two possible reasons. The selective reporting of certain analyses with statistically significant results is another likely source of reporting bias [44, 46, 47]. Each of the research areas we examined is likely to be linked to variable levels of reporting bias due to the different economics, dynamics, and conflicts of interest in each discipline [64, 65]. Interpreting the literature as a whole is challenging, considering the numerous biases that may affect the reliability and integrity of the scientific enterprise [66, 67].

To obtain a complete picture of the evidence (i.e., without reporting bias), it is important to know the results from all conducted studies on a given research question [68]. In our study, results from meta-analyses of health-related behavior and cardiovascular disease mortality and all-cause mortality were more likely to be affected by reporting bias compared to statin meta-analyses (22% and 24% versus 0%, respectively). The literature of health-related behavior is almost exclusively composed by observational studies, whereas statins are most often studied using randomized controlled trials. Reporting bias may be less frequent among trials than observational studies because several efforts to increase transparency and reproducibility of results have been adopted over the history of randomized controlled trials [69]. These include the mandatory registration of all clinical trials in humans and disclosure of all results [70]. As of more recently, data sharing statements of clinical trials are also required [71]. Observational epidemiologic studies should embrace these reproducible research practices to reduce reporting bias in the literature [68–70, 72]. These practices could involve key elements of the scientific process, including (a) methods (e.g., rigorous training in statistics), (b) reporting and dissemination (e.g., disclosure of conflicts of interest), (c) reproducibility (e.g., open data), (d) evaluation (e.g., pre- and postpublication peer review), and (e) incentives (e.g., funding replication studies) [72]. Improving methodological training involves aspects of both research design and statistical analyses—for example, correct interpretation of *P* values [73], acknowledging the importance of statistical power, and improving the accuracy of effect sizes [72]. Protecting against cognitive biases is another major issue that has been overlooked [72]. Protecting against conflict of interests, especially financially related, is an imperative to achieve reproducible science. In addition to disclosure of potential conflicts of interest, promoting preregistration of study procedures and analytical plan may prevent reporting bias favoring positive results [72]. Funding replication of studies and encouraging openness in science and reproducibility practices by making datasets, scripts, and software publicly available may increase transparency and credibility of scientific claims [72]. For instance, food industry–sponsored studies are more likely to report conclusions favorable to the sponsors [74] but frequently lack transparency on acknowledgment of the funding source [75]. Further examples of reproducibility practices have been described and discussed by Munafò and colleagues [72].

To our knowledge, our analysis is the first comparative assessment of reporting bias across different fields of health-related behavior and statins. Our findings were based on well-established statistical tests developed to detect different aspects of reporting bias, as well as a complementary assessment of the risk of bias of systematic reviews using the ROBIS tool. We selected the ROBIS tool as it has greater specification to assess risk of bias compared to other tools. For instance, the “Assessing the Methodological Quality of Systematic Reviews” (AMSTAR) that has been used to evaluate the methodological quality of systematic reviews has constructs that are more related to quality of reporting than risk of bias [76, 77]. Risk of bias is linked to methodological quality of systematic reviews but provides further evaluation on how methodological limitations were considered to form conclusions. In this sense, the ROBIS tool is increasingly being used to assess risk of bias not only in systematic reviews [41, 76, 78] but also in guideline committees that evaluate evidence level (e.g., Australian government, National Health and Medical Research Council). Our ROBIS tool results showed that most of the systematic reviews had high risk of bias. Similar findings have been observed in previous studies appraising risk of bias in other research areas using the ROBIS tool [76, 78]. For instance, 18 (58%) out of 31 systematic reviews evaluating the effectiveness of intra-articular hyaluronic acid injection in treating knee osteoarthritis had high (*n* = 16) or unclear (*n* = 2) risk of bias [78]. Another survey assessing systematic reviews about psoriasis found that most reviews (86%) were classified as high risk of bias [76]. It is noteworthy that high risk of bias was found even for systematic reviews exhibiting high methodological quality as assessed through AMSTAR [76].

Our ROBIS assessment indicated that identification and selection of studies (i.e., appropriate range of databases, terms and filters used, and efforts to minimize errors in selection of studies) are major concerns. These biases in the review process could explain, at least in part, reporting bias results obtained from small-study effect and excess significance tests. The synthesis and findings domain also revealed potential risk of bias due to insufficient inclusion of studies and appropriate synthesis of estimates. This domain also reflects between-study variation, robustness of findings (e.g., sensitivity analyses), and biases in synthesis findings (i.e., if evaluated by systematic reviews).

We used small-study effect and excess significance tests to appraise reporting bias in the literature, which are the most commonly recommended and used methods [79]. However, results from these tests might also reflect methodological and clinical heterogeneity, or even chance [42]. In fact, most meta-analyses contained moderate to high heterogeneity (based on I^2^ statistic; S4 Table). Results from an Egger test (small-study effect) can give spurious false positive results due to correlation between log of effect size and its variance, especially in the presence of heterogeneity between studies in a meta-analysis. An alternative better-performing test has been proposed by Peters to identify reporting bias in meta-analyses, but it requires data from a 2 × 2 table [80]. Such data were rarely reported in individual studies in the meta-analyses of observational studies. As also noted by Tsilidis and colleagues [81], meta-analyses commonly use maximally adjusted relative risks rather than unadjusted relative risks calculated from 2 × 2 tables. For such data, the use of the Egger test is appropriate.

The egger test and excess significance test have low power to detect reporting bias and do not give indication about what the sources of bias are. Therefore, we performed sensitivity analyses, retaining only meta-analyses with ≥10 primary studies. In this subsample of meta-analyses, evidence of reporting bias was higher than the entire sample (small-study effect: 31% versus 18%; excess significance: 27% versus 20%). Differences between primary results and sensitivity analyses are likely related to low power of reporting bias tests, which could lead to false negative results in the former group of meta-analyses. Therefore, our estimates of reporting bias in the meta-analyses are possibly conservative. The ranking of research areas according to levels of reporting bias was also different between the main analysis and the sensitivity analysis (i.e., meta-analyses with ≥10 primary studies). For instance, meta-analyses of sedentary behavior appeared most sensitive to this restriction, as the estimated proportion of reporting bias increased when calculated with either the small-study effects (from 9% to 50%) or excess significance tests (from 18% to 100%). A possible explanation could be the small fraction of meta-analyses with ≥10 primary studies (2 out of 12) in this relatively new research field [82].

It is important to acknowledge that certain methodological decisions we made may have introduced bias in the sample of reviews selected or may compromise the generalizability of our findings. We excluded systematic reviews on alcohol published in Chinese language (*n* = 2), which potentially have high risk of bias [83]. In addition, we restricted our analyses to systematic reviews published in this decade only (2010–2016), which explains the small number of included meta-analyses in some research areas. This may have limited comparisons of the extent of reporting bias between research areas investigated. Our results may not provide a complete historical assessment of reporting bias in these areas. Nevertheless, our results reflect reporting bias in the literature of recent and relevant public health topics and from a time period when reporting standards have been improving due to, e.g., the widespread use of various manuscript reporting checklists [84]. Recent systematic reviews contain a higher number of primary studies than older systematic reviews and synthesize evidence of emerging fields that have flourished only recently (i.e., sedentary behavior).

In conclusion, we found evidence of reporting bias in approximately one-fifth of recent meta-analyses of observational studies of health-related behavior (physical activity, sedentary behavior, smoking, alcohol consumption, diet) and cardiovascular and all-cause mortality. Such a level of reporting bias may, to some extent at least, distort conclusions arising from this body of evidence. Contrarily, we found no evidence of reporting bias in meta-analyses of randomized controlled trials of statins.

## Materials and methods

### Identification and selection of relevant systematic reviews

We searched Medline (through PubMed), Embase (i.e., excluding Medline), Cochrane Methodology Register Database, and Web of Science for systematic reviews published between 2010 and 2016. We restricted our search to recent systematic reviews for several reasons. These systematic reviews belong to a “birth cohort” of systematic reviews published after the launch of the Meta-analysis of Observational Studies in Epidemiology (MOOSE) [85] and Preferred Reporting Items for Systematic Reviews and Meta-Analyses (PRISMA) [86] guidelines and are expected to have lower risk of bias. As we were interested in comparing levels of bias across different research areas, this restriction may have reduced confounding due to date of publication. We restricted the search, as well as the successive phases of our study, to systematic reviews aiming to investigate the associations of health-related behavior (tobacco, alcohol, diet [fat, fruits and vegetables, salt, and sugar], physical activity, and sedentary behavior) and statins with cardiovascular disease mortality (overall cardiovascular mortality and cause-specific deaths from cardiovascular disease) and all-cause mortality. We accepted any definition for the exposures and the outcomes as defined in the original systematic reviews. The keywords used in the search are described in S1 Text, and files exported from databases during search strategy with all studies screened and selected are available at https://osf.io/wpb69/.

Systematic reviews were screened and selected (by two reviewers, and disagreements solved by a third reviewer) based on the following eligibility criteria: (i) sought to investigate an exposure–outcome association in a nonclinical population; (ii) systematically searched for primary studies and performed a meta-analysis (i.e., weighted summary effect size) using results from primary studies; (iii) selected only observational studies (cohort and case-control studies) if a health-related behavior review and only randomized controlled trials if a statin review; (v) reported data from each primary study included in the meta-analysis (S1 Text).

We decided a priori that a random sample of up to 20 systematic reviews per research area (tobacco, alcohol, diet, physical activity, sedentary behavior, and statins) would be included to compare levels of reporting bias in the relevant literature. If our search retrieved fewer than 20 meta-analyses in a given research area, we included them all. A similar study-selection strategy was recently used in a study evaluating publication bias in meta-analyses of individual studies [87]. These methods were decided a priori as described in the analysis plan available at https://osf.io/wpb69/ (not published prior to the identification and selection of systematic reviews).

### Risk of bias in systematic reviews

Reporting bias could be related to overall risk of bias in a review. Therefore, four reviewers (JPRL, NC, AF, LP), working in pairs, independently assessed the risk of bias in the included systematic reviews using the ROBIS tool [41]. ROBIS comprises three phases: (1) assess relevance; (2) identify concerns with review process; (3) judge risk of bias in the review. To assess relevance, we extracted the target question from each review using the PICOS acronym (participants, interventions, comparisons, outcomes) or equivalents for etiological questions (participants, exposure, comparisons, outcomes). In phase 2, we assessed the risk of bias in four domains related to the review process: (1) study eligibility criteria; (2) identification and selection of studies; (3) data collection and study appraisal; and (4) synthesis and findings. Questions included in each of the four domains are available in S3 Table. Questions were answered as “Yes,” “Probably Yes,” “Probably No,” “No,” and “No Information,” with “Yes” indicating low risk of bias. In phase 3, we summarized the concerns identified in each domain during phase 2 and risk of bias in the review as low, high, or unclear. Further details on the ROBIS tool are described elsewhere [41].

### Risk of reporting bias in the body of evidence

For each meta-analysis performed in the selected systematic reviews, we assessed the extent of reporting bias in the included literature via small-study effect [42] and excess significance tests [88]. To perform these tests, we extracted necessary data (e.g., effect size, confidence intervals, sample size, and number of events [deaths]) for each primary study included in the main meta-analysis performed in the systematic reviews. We also used these data to reperform the meta-analyses (i.e., using random effect models, which was used in the majority of the original meta-analyses). We did this to describe the number of meta-analyses with nominally statistically significant results at *P* < 0.05 (S1 Text).

Small-study effect test (also known as regression asymmetry test, proposed by Egger and colleagues) evaluates whether smaller studies tend to overestimate the effect size estimates compared to larger studies. For this matter, the test evaluates whether the association between effect size (e.g., relative risk, odds ratio) and precision (standard error) is greater than might be expected by chance. We considered a *P* value < 0.10 as a statistical significance threshold for small-study effect bias (i.e., suggesting evidence of reporting bias), as initially proposed by Egger and colleagues [42, 89] and consistently used in the literature [42, 66, 81, 87, 90, 91].

Excess significance test evaluates whether the O differs from the E. The E in each meta-analysis was obtained from the sum of power estimates of each primary study. The power estimate of each primary study depends on the plausible causal effect of each research area (e.g., smoking and cardiovascular mortality), which was assumed to be the effect of the most precise primary study (smaller standard error) in each meta-analysis [88]. We considered *P* < 0.10 (one-side *P* < 0.05 for O > E) as a statistical significance threshold for excess significance bias [43, 88]. The excess significance is reported as a proportion of studies, with the higher proportion indicating more excess significance (O > E) and thus more evidence of reporting bias.

Due to the low power of these bias tests, we performed a sensitivity analysis excluding meta-analyses with fewer than 10 studies to analyze the impact in the results. We also performed a sensitivity analysis excluding small individual studies (fewer than 200 deaths) within meta-analyses to evaluate whether results reflect reporting bias among small studies only. We performed all statistical analyses using Stata version 15.0 (College Station, TX).

## Supporting information

S1 Data Raw data of figures. (XLSX)

S1 Text Supporting materials and methods. (DOC)

S1 Table List of excluded studies and reasons for exclusion, by research area. (DOC)

S2 Table Summary of reasons for excluding studies during full-text screening, by research area. (DOC)

S3 Table Risk of bias in systematic reviews of physical activity, sedentary behavior, diet, alcohol, smoking, and statins, using ROBIS tool. (DOC)

S4 Table Meta-analyses of cardiovascular disease and all-cause mortality, by research area. (DOC)

S5 Table Sensitivity analysis excluding individual studies with fewer than 200 deaths. Relative and absolute frequencies of meta-analyses with nominally statistically significant results, small-study effects, and excess significance, by research area.
(DOC)

## Abbreviations

AMSTAR: Assessing the Methodological Quality of Systematic Reviews
CHD: coronary heart disease
DHA: docosahexaenoic acid
E: number of expected primary studies with *P* < 0.05 results
EPA: eicosapentaenoic acid
IHD: ischemic heart disease
MI: myocardial infarction
MOOSE: Meta-analysis of Observational Studies in Epidemiology
MUFA: monounsaturated fatty acid
O: number of observed primary studies with *P* < 0.05 results
PRISMA: Preferred Reporting Items for Systematic Reviews and Meta-Analyses
PUFA: polyunsaturated fatty acid
SFA: saturated fatty acid
