# Supplementary material for: Reporting bias in the literature on the associations of health-related behaviors and statins with cardiovascular disease and all-cause mortality
Source: PLoS Biol. 2018 Jun 18;16(6):e2005761. doi: 10.1371/journal.pbio.2005761 (PMC6023226; doi:10.1371/journal.pbio.2005761)
Supplement: S1 Text — (DOC) [file pbio.2005761.s002.doc]

**S1 Text – Supporting Material and Methods**

We searched Medline, Embase, Cochrane Methodology Register Database, PsycINFO, and Web of Science for systematic reviews published between 2010 and 2016. We restricted our search to recent systematic reviews for several reasons. These systematic reviews belong to a “birth cohort” of systematic reviews published after the launch of the PRISMA (Preferred Reporting Items for Systematic Reviews and Meta-Analyses) and are expected to have lower risk of bias. As we were interested in comparing levels of reporting bias across different research areas, this restriction may have reduced confounding due to date of publication.

We restricted the search, as well as the successive phases of our study, on systematic reviews aiming to investigate the associations of key health-related behaviors [physical activity, and sedentary behavior, alcohol, smoking, diet (fat, fruits and vegetables, salt, and sugar)] and statins with cardiovascular and all-cause mortality. We used the following keywords to search the literature, filtering by study design (“systematic reviews” AND “meta-analysis”):

**Physical Activity:**

(((“physical inactivity” OR “physical activity” OR motor activity OR “physical exercise” OR exercise OR MVPA OR walking OR cycling OR “aerobic exercise”))) AND (((death) OR cardiovascular mortality) OR all-cause mortality);

**Sedentary Behavior:**

(((sedentary behavior OR sedentary behaviour OR sedentary lifestyle OR “sedentary time” OR “sitting time” OR “television viewing” OR “TV” OR “screen time” OR driving OR "screen based" OR “video game” OR computer))) AND (((death) OR cardiovascular mortality) OR all-cause mortality);

**Alcohol Intake:**

((“ethanol” OR “alcohol” OR “alcoholic beverages” OR “drinking behaviour” OR “alcohol drinking” OR “drink*” OR “liquor*” OR “ethanol intake” OR “alcohol* drink*” OR “ethanol drink*”)) AND (((death) OR cardiovascular mortality) OR all-cause mortality);

**Smoking:**

((tobacco OR smoking OR cigarette)) AND (((death) OR cardiovascular mortality) OR all-cause mortality);

**Diet:**

*Fat Intake:* (((dietary fat OR omega 6 OR omega 3 OR fat intake OR fat OR saturated fat OR trans-fat OR monounsaturated fat OR polyunsaturated))) AND (((death) OR cardiovascular mortality) OR all-cause mortality)

*Sugar Intake:* ((“sugar-sweetened beverages” OR "sugar*" OR sucrose OR fructose OR "dietary sucrose" OR "soft drink*" OR "refined sugar")) AND (((death) OR cardiovascular mortality) OR all-cause mortality).

*Salt Intake:* ((Salt intake OR sodium intake OR na intake OR high salt diet)) AND (((death) OR cardiovascular mortality) OR all-cause mortality)

*Fruit and Vegetables:* ((Fruit OR Citrus OR Vegetables OR fruit* OR vegetable* OR orange* OR apple* OR pear OR pears OR grape or grapes OR banana* OR berry or berries OR citrus OR carrot* OR greens OR cabbage* OR brassica* OR blackberr* OR blueberr* OR cranberr* OR guava* OR kiwi* OR lingonberr* OR mango* OR melon* OR papaya* OR pineapple* OR raspberr* OR strawberr* OR tomato* OR potato* OR onion* OR grapefruit* OR mandarin* OR satsuma* OR tangerine* OR plum OR plums OR apricot* OR cherry OR cherries OR nectarine* OR peach OR peaches)) AND (((death) OR cardiovascular mortality) OR all-cause mortality);

**Statin:**

((statins OR statin OR “lipid lowering” OR Pravastatin OR Atorvastatin OR Lipitor OR Torvast OR Fluvastatin OR “Hydroxymethylglutaryl-CoA Reductase Inhibitors" OR Simvastatin OR Rosuvastatin OR Lovastatin OR Mevastatin OR Cerivastatin)) AND (((death) OR cardiovascular mortality) OR all-cause mortality).

We imported all the studies retrieved into the EndNote X7 to remove duplicates. Two reviewers independently (LFMR and JPRL) examined the title and abstract of all records and disagreements were settled by a third reviewer (THS). The same scheme was used to check for the eligibility criteria in the full-text of the selected records in the previous stage.

To be included in the final sample systematic reviews had to meet the following eligibility criteria:

1. Sought to investigate an exposure-outcome association in a general healthy adult population. Exposures-outcome associations were restricted to studies on (physical activity OR sedentary behavior OR alcohol OR smoking OR diet OR statins) AND (cardiovascular mortality OR all-cause mortality). We excluded reviews of prognostic studies with diseased population. For statins only, we included systematic reviews of adult’s population with CVD risk factors, but not for those with history of CVD. Use of statins is recommend for the primary prevention in adults with the following conditions: 1) 40 to 75 years; 2) One or more CVD risk factors (*i.e.*, dyslipidemia, diabetes, hypertension, or smoking); 3) with calculated 10-year risk of a cardiovascular event of 10% or greater (<https://www.uspreventiveservicestaskforce.org/Page/Document/draft-recommendation-statement175/statin-use-in-adults-preventive-medication1>)
2. Searched individual studies through a systematic-search of literature and performed a meta-analysis (*i.e.*, weighted summary effect size) using results from individual studies; We excluded narrative reviews, systematic reviews without meta-analysis, network meta-analysis, and individual patient data meta-analyses because these sorts of reviews did not provide data required to perform tests to identify bias in the body of the evidence.
3. Selected only observational studies (cohort and case-control studies) if a health-related behavior meta-analysis, otherwise only randomized controlled trials if a statins meta-analysis; Since RCT cannot always be ethically or logistically conducted, we restricted to systematic reviews of observational studies assessing associations between health-related behavior and cardiovascular and all-cause mortality. On the other hand, meta-analyses for statins were restricted to RCT.
4. Reported data from each individual study included in the meta-analysis; Several data (see data extraction section below) regarding the primary studies included in the meta-analyses are needed to evaluate the risk of bias the body of evidence via small study effects and excess significance tests. We excluded systematic reviews that did not report at least the maximally adjusted effect size with its respective 95% CI for each primary study included in the main meta-analysis.

We included only systematic reviews in English, Portuguese, and Spanish language.

**Data extraction**

For each systematic review, we extracted the following information: first author, year of publication, exposure-outcome association, number of included studies, sum of total sample size and number of events (sum of all primary studies included) and weighted summary effect size with its 95% confidence intervals (95% CI).

We also extracted the following information from each primary study included in the meta-analyses: study design (RCT, cohort, or case-control), number of events and total sample size (for cohort and RCT studies), number of cases and controls (for case-control studies), maximally adjusted effect size (reported as odds ratio for case-control studies and hazard ratio or mortality ratio for cohort and RCT) with its respectively 95% CI and P values. To obtain these data we first searched in the systematic review. If these data were not available, we contacted the first author of the systematic review and, if necessary, extracted data from the original studies. In case of lack of clarity in the information presented in a meta-analysis, authors were directly contacted to resolve any unclear points. Data extraction was performed by trained research assistants and reviewed by one investigator (LR).

**Data Analysis**

We re-performed each meta-analysis (*i.e.*, using random effect models) conducted in the systematic reviews in order to estimate summary effect measures and its 95% confidence intervals. We included only one estimate per primary study in the meta-analysis. Whenever effects were not available for the total sample size of the primary study (*e.g.*, relative risks and 95% CI were provided separated by sex), we performed a meta-analysis using fixed effect models within stratum-categories. Finally, RR and 95% CI from fixed effect models were included in the meta-analysis.
