# Supplementary material for: Reporting bias in the literature on the associations of health-related behaviors and statins with cardiovascular disease and all-cause mortality
Source: PLoS Biol. 2018 Jun 18;16(6):e2005761. doi: 10.1371/journal.pbio.2005761 (PMC6023226; doi:10.1371/journal.pbio.2005761)
Supplement: S1 Table — (DOC) [file pbio.2005761.s003.doc]

**S1 Table**: List of excluded studies and reasons for exclusion, by research area.

| Reference | Reason for exclusion |
| --- | --- |
| ***Physical activity*** |  |
| Dahabreh IJ, Paulus JK. Association of episodic physical and sexual activity with triggering of acute cardiac events: systematic review and meta-analysis. JAMA 2011;305(12):1225-33. doi: 10.1001/jama.2011.336 | Other exposure |
| Kivimaki M, Nyberg ST, Fransson EI, et al. Associations of job strain and lifestyle risk factors with risk of coronary artery disease: a meta-analysis of individual participant data. CMAJ. 2013;185(9):763-9. doi: 10.1503/cmaj.121735 | Other exposure |
| Zhang Y, Hu G. Dietary Pattern, Lifestyle Factors, and Cardiovascular Diseases. Current Nutrition Reports 2012;1(2):64-72. | Other exposure |
| Ekelund U, Steene-Johannessen J, Brown WJ, et al. Does physical activity attenuate, or even eliminate, the detrimental association of sitting time with mortality? A harmonised meta-analysis of data from more than 1 million men and women. The Lancet 2016;388(10051):1302-10. | Other exposure |
| Reimers CD, Knapp G, Reimers AK. Does physical activity increase life expectancy? A review of the literature. Journal of Aging Research 2012;2012(243958) | Not systematic review |
| Oja P, Titze S, Bauman A, et al. Health benefits of cycling: a systematic review. Scandinavian Journal of Medicine & Science in Sports 2011;21(4):496-509. doi: 10.1111/j.1600-0838.2011.01299.x | Data Extraction: did not provide data from individual studies or did not perform meta-analysis |
| Li J, Loerbroks A, Angerer P. Physical activity and risk of cardiovascular disease: what does the new epidemiological evidence show? Current Opinion in Cardiology 2013;28(5):575-83. doi: 10.1097/HCO.0b013e328364289c | Other outcome |
| Koba S, Tanaka H, Maruyama C, et al. Physical Activity in the Japan Population: Association with Blood Lipid Levels and Effects in Reducing Cardiovascular and All-Cause Mortality. Journal of Atherosclerosis and Thrombosis 2011;18(10):833-45. | Not systematic review |
| Fogelholm M. Physical activity, fitness and fatness: relations to mortality, morbidity and disease risk factors. A systematic review. Obesity Reviews 2010;11(3):202-21. doi: 10.1111/j.1467-789X.2009.00653.x | Data Extraction: did not provide data from individual studies or did not perform meta-analysis |
| Hartley L, Lee MS, Kwong JSW, et al. Qigong for the primary prevention of cardiovascular disease. Cochrane Database of Systematic Reviews 2015(6) doi: 10.1002/14651858.CD010390.pub2 | Other outcome |
| Milton K, Macniven R, Bauman A. Review of the epidemiological evidence for physical activity and health from low-and middle-income countries. Global Public Health 2014;9(4):369-81. doi: 10.1080/17441692.2014.894548 | Data Extraction: did not provide data from individual studies or did not perform meta-analysis |
| Zheng G, Huang M, Liu F, et al. Tai Chi Chuan for the primary prevention of stroke in middle-aged and elderly adults: A systematic review. Evidence-based Complementary and Alternative Medicine 2015;2015(742152) | Other outcome |
| Hartley L, Flowers N, Lee MS, et al. Tai chi for primary prevention of cardiovascular disease. Cochrane Database of Systematic Reviews 2014; (4). http://onlinelibrary.wiley.com/doi/10.1002/14651858.CD010366.pub2/abstract. | Other outcome |
| Goodman JM, Burr JF, Banks L, et al. The Acute Risks of Exercise in Apparently Healthy Adults and Relevance for Prevention of Cardiovascular Events. Canadian Journal of Cardiology 2016;32(4):523-32. doi: 10.1016/j.cjca.2016.01.019 | Other exposure |
| Yerrakalva D, Mullis R, Mant J. The associations of "fatness," "fitness," and physical activity with all-cause mortality in older adults: A systematic review. Obesity 2015;23(10):1944-56. doi: 10.1002/oby.21181 | Other exposure |
| Liu B, Hu X, Zhang Q, et al. Usual walking speed and all-cause mortality risk in older people: A systematic review and meta-analysis. Gait and Posture 2016;44:172-77. | Other exposure |
| Hartley L, Dyakova M, Holmes J, et al. Yoga for the primary prevention of cardiovascular disease. Cochrane Database Syst Rev 2014;5:Cd010072. doi: 10.1002/14651858.CD010072.pub2 [published Online First: 2014/05/16] | Other outcome |
| ***Sedentary behavior*** |  |
| Rhodes RE, Mark RS, Temmel CP. Adult sedentary behavior: a systematic review. Am J Prev Med 2012;42(3):e3-28. doi: 10.1016/j.amepre.2011.10.020 | Other outcome |
| Rezende LFM, de Sa TH, Mielke GI, et al. All-Cause Mortality Attributable to Sitting Time Analysis of 54 Countries Worldwide. American Journal of Preventive Medicine 2016;51(2):253-63. doi: 10.1016/j.amepre.2016.01.022 | Not systematic review |
| Kivimaki M, Nyberg ST, Fransson EI, et al. Associations of job strain and lifestyle risk factors with risk of coronary artery disease: a meta-analysis of individual participant data. CMAJ : Canadian Medical Association journal = journal de l'Association medicale canadienne 2013;185(9):763-9. doi: 10.1503/cmaj.121735 | Other exposure |
| Ekelund U, Steene-Johannessen J, Brown WJ, et al. Does physical activity attenuate, or even eliminate, the detrimental association of sitting time with mortality? A harmonised meta-analysis of data from more than 1 million men and women. Lancet (London, England) 2016;388(10051):1302-10. doi: 10.1016/s0140-6736(16)30370-1 | Data Extraction: did not provide data from individual studies or did not perform meta-analysis |
| van Uffelen JG, Wong J, Chau JY, et al. Occupational sitting and health risks: a systematic review. Am J Prev Med 2010;39(4):379-88. doi: 10.1016/j.amepre.2010.05.024 | Data Extraction: did not provide data from individual studies or did not perform meta-analysis |
| Rezende LF, Rey-Lopez JP, Rodrigues Matsudo VK, et al. Sedentary behavior and health outcomes among older adults: a systematic review. BMC public health 2014;14 doi: 10.1186/1471-2458-14-333 | Data Extraction: did not provide data from individual studies or did not perform meta-analysis |
| Proper KI, Singh AS, van Mechelen W, et al. Sedentary behaviors and health outcomes among adults: a systematic review of prospective studies. Am J Prev Med 2011;40(2):174-82. doi: 10.1016/j.amepre.2010.10.015 | Data Extraction: did not provide data from individual studies or did not perform meta-analysis |
| Thorp AA, Owen N, Neuhaus M, et al. Sedentary behaviors and subsequent health outcomes in adults a systematic review of longitudinal studies, 1996-2011. Am J Prev Med 2011;41(2):207-15. doi: 10.1016/j.amepre.2011.05.004 | Data Extraction: did not provide data from individual studies or did not perform meta-analysis |
| Biddle SJ, Bennie JA, Bauman AE, et al. Too much sitting and all-cause mortality: is there a causal link? BMC public health 2016;16:635. doi: 10.1186/s12889-016-3307-3 | Not systematic review |
| ***Alcohol*** |  |
| Liu PM, Dosieah S, Zheng HS, et al. [Alcohol consumption and coronary heart disease in Eastern Asian men: a meta-analysis of prospective cohort studies]. Zhonghua xin xue guan bing za zhi 2010;38(11):1038-44 | Chinese language |
| Liu PM, Dosieah S, Luo NS, et al. [Alcohol intake and stroke in Eastern Asian men:a systemic review and meta-analysis of 17 prospective cohort studies]. Zhonghua yi xue za zhi 2010;90(40):2834-8. | Chinese language |
| Costanzo S, Di Castelnuovo A, Donati MB, et al. Alcohol Consumption and Mortality in Patients With Cardiovascular Disease A Meta-Analysis. Journal of the American College of Cardiology 2010;55(13):1339-47. | Clinical population |
| Jin M, Cai S, Guo J, et al. Alcohol drinking and all cancer mortality: a meta-analysis. Annals of Oncology 2013;24(3):807-16. doi: 10.1093/annonc/mds508 | Other outcome |
| Branas CC, Han S, Wiebe DJ. Alcohol Use and Firearm Violence. Epidemiologic Reviews 2016;38(1):32-45. doi: 10.1093/epirev/mxv010 | Other outcome |
| Roerecke M, Rehm J. Alcohol use disorders and mortality: a systematic review and meta-analysis. Addiction 2013;108(9):1562-78. doi: 10.1111/add.12231 | Clinical population |
| Darvishi N, Farhadi M, Haghtalab T, et al. Alcohol-Related Risk of Suicidal Ideation, Suicide Attempt, and Completed Suicide: A Meta-Analysis. Plos One 2015;10(5) doi: 10.1371/journal.pone.0126870 | Other outcome |
| Huang C, Zhan J, Liu YJ, et al. Association between alcohol consumption and risk of cardiovascular disease and all-cause mortality in patients with hypertension: a meta-analysis of prospective cohort studies. Mayo Clinic proceedings 2014;89(9):1201-10. doi: 10.1016/j.mayocp.2014.05.014 | Clinical population |
| McQueen J, Howe TE, Allan L, et al. Brief interventions for heavy alcohol users admitted to general hospital wards. Cochrane Database of Systematic Reviews 2011;(8).http://onlinelibrary.wiley.com/doi/10.1002/14651858.CD005191.pub3/abstract. | Clinical population |
| Fernandez-Sola J. Cardiovascular risks and benefits of moderate and heavy alcohol consumption. Nature Reviews Cardiology 2015;12(10):576-87. doi: 10.1038/nrcardio.2015.91 | Not systematic review |
| Crippa A, Discacciati A, Larsson SC, et al. Coffee consumption and mortality from all causes, cardiovascular disease, and cancer: a dose-response meta-analysis. American journal of epidemiology 2014;180(8):763-75. doi: 10.1093/aje/kwu194 | Other exposure |
| Zhang Y, Hu G. Dietary Pattern, Lifestyle Factors, and Cardiovascular Diseases. Current Nutrition Reports 2012;1(2):64-72. doi: 10.1007/s13668-012-0009-z | Not systematic review |
| Zhang XY, Shu L, Si CJ, et al. Dietary Patterns, Alcohol Consumption and Risk of Coronary Heart Disease in Adults: A Meta-Analysis. Nutrients 2015;7(8):6582-605. doi: 10.3390/nu708530 | Other outcome |
| Calabria B, Degenhardt L, Hall W, et al. Does cannabis use increase the risk of death? Systematic review of epidemiological evidence on adverse effects of cannabis use. Drug and alcohol review 2010;29(3):318-30. doi: 10.1111/j.1465-3362.2009.00149.x | Other exposure |
| Wang C, Xue H, Wang Q, et al. Effect of drinking on all-cause mortality in women compared with men: a meta-analysis. Journal of women's health (2002) 2014;23(5):373-81. doi: 10.1089/jwh.2013.4414 | Data Extraction: did not provide data from individual studies or did not perform meta-analysis |
| Probst C, Roerecke M, Behrendt S, et al. Gender differences in socioeconomic inequality of alcohol-attributable mortality: A systematic review and meta-analysis. Drug and alcohol review 2015;34(3):267-77. doi: 10.1111/dar.12184 | Other exposure |
| McCambridge J, Hartwell G. Has industry funding biased studies of the protective effects of alcohol on cardiovascular disease? A preliminary investigation of prospective cohort studies. Drug and alcohol review 2015;34(1):58-66. doi: 10.1111/dar.12125 | Not systematic review |
| Hansel B, Kontush A, Bruckert E. Is a cardioprotective action of alcohol a myth? Current Opinion in Cardiology 2012;27(5):550-55. doi: 10.1097/HCO.0b013e328356dc30 | Other outcome |
| Rehm J, Shield KD, Roerecke M, et al. Modelling the impact of alcohol consumption on cardiovascular disease mortality for comparative risk assessments: an overview. BMC public health 2016;16 doi: 10.1186/s12889-016-3026-9 | Not systematic review |
| Colpani V, Baena C, Jaspers L, et al. Modifiable risk factors for prevention of cardiovascular disease and mortality in middle-aged women: Systematic review and meta-analysis. Circulation 2015;131 | Abstract only |
| Uthman OA, Hartley L, Rees K, et al. Multiple risk factor interventions for primary prevention of cardiovascular disease in low- and middle-income countries. Cochrane Database of Systematic Reviews 2015; (8). http://onlinelibrary.wiley.com/doi/10.1002/14651858.CD011163.pub2/abstract. | Other outcome |
| Grosso G, Yang J, Marventano S, et al. Nut consumption on all-cause, cardiovascular, and cancer mortality risk: a systematic review and meta-analysis of epidemiologic studies. American Journal of Clinical Nutrition 2015;101(4):783-93. doi: 10.3945/ajcn.114.099515 | Other outcome |
| Roerecke M, Gual A, Rehm J. Reduction of Alcohol Consumption and Subsequent Mortality in Alcohol Use Disorders: Systematic Review and Meta-Analyses. Journal of Clinical Psychiatry 2013;74(12):E1181-U102. doi: 10.4088/JCP.13r08379 | Clinical population |
| Laramee P, Leonard S, Buchanan-Hughes A, et al. Risk of All-Cause Mortality in Alcohol-Dependent Individuals: A Systematic Literature Review and Meta-Analysis. Ebiomedicine 2015;2(10):1394-404. doi: 10.1016/j.ebiom.2015.08.040 | Clinical population |
| Shield KD, Rehm J. Russia-specific relative risks and their effects on the estimated alcohol-attributable burden of disease. BMC public health 2015;15 doi: 10.1186/s12889-015-1818-y | Not systematic review |
| Roerecke M, Rehm J. The cardioprotective association of average alcohol consumption and ischaemic heart disease: a systematic review and meta-analysis. Addiction 2012;107(7):1246-60. doi: 10.1111/j.1360-0443.2012.03780.x | Data Extraction: did not provide data from individual studies or did not perform meta-analysis |
| ***Smoking*** |  |
| Mons U, Muezzinler A, Gellert C, et al. Impact of smoking and smoking cessation on cardiovascular events and mortality among older adults: meta-analysis of individual participant data from prospective cohort studies of the CHANCES consortium. Bmj-British Medical Journal 2015;350 doi: 10.1136/bmj.h1551 | Data Extraction: did not provide data from individual studies or did not perform meta-analysis |
| Murakami Y. Meta-analyses using individual participant data from cardiovascular cohort studies in Japan: current status and future directions. Journal of epidemiology / Japan Epidemiological Association 2014;24(2):96-101. | Data Extraction: did not provide data from individual studies or did not perform meta-analysis |
| Colpani V, Baena C, Jaspers L, et al. Modifiable risk factors for prevention of cardiovascular disease and mortality in middle-aged women: Systematic review and meta-analysis. Circulation 2015;131 | Abstract only |
| Woodcock J, Franco OH, Orsini N, et al. Non-vigorous physical activity and all-cause mortality: Systematic review and meta-analysis of cohort studies. International Journal of Epidemiology 2011;40(1):121-38. | Other exposure |
| Sinha DN, Palipudi KM, Gupta PC, et al. Smokeless tobacco use: A meta-analysis of risk and attributable mortality estimates for India. Indian Journal of Cancer 2014;51(5):73-77. doi: 10.4103/0019-509x.147477 | Not systematic review |
| Silva Vde L, Cesse EA, de Albuquerque Mde F. Social determinants of death among the elderly: a systematic literature review. Revista brasileira de epidemiologia. 2014;17 Suppl 2:178-93 | Other exposure |
| Chang CM, Corey CG, Rostron BL, et al. Systematic review of cigar smoking and all cause and smoking related mortality. BMC public health 2015;15 doi: 10.1186/s12889-015-1617-5 | Data Extraction: did not provide data from individual studies or did not perform meta-analysis |
| Lee PN. The effect of reducing the number of cigarettes smoked on risk of lung cancer, COPD, cardiovascular disease and FEV1 - A review. Regulatory Toxicology and Pharmacology 2013;67(3):372-81. doi: 10.1016/j.yrtph.2013.08.016 | Not systematic review |
| Waziry R, Jawad M, Ballout RA, et al. The effects of waterpipe tobacco smoking on health outcomes: an updated systematic review and meta-analysis. Int J Epidemiol 2016 doi: 10.1093/ije/dyw021 | Other outcome |
| ***Diet*** |  |
| Aburto NJ, Ziolkovska A, Hooper L, et al. Effect of lower sodium intake on health: Systematic review and meta-analyses. BMJ (Online) 2013;346(7903) | Data Extraction: did not provide data from individual studies or did not perform meta-analysis |
| Schwab U, Lauritzen L, Tholstrup T, et al. Effect of the amount and type of dietary fat on cardiometabolic risk factors and risk of developing type 2 diabetes, cardiovascular diseases, and cancer: a systematic review. Food & nutrition research 2014;58 doi: 10.3402/fnr.v58.25145 | Data Extraction: did not provide data from individual studies or did not perform meta-analysis |
| Harcombe Z, Baker JS, Davies B. Evidence from prospective cohort studies did not support the introduction of dietary fat guidelines in 1977 and 1983: a systematic review. Br J Sports Med 2016 doi: 10.1136/bjsports-2016-096409 | Data Extraction: did not provide data from individual studies or did not perform meta-analysis |
| Grosso G, Marventano S, Yang J, et al. A Comprehensive Meta-analysis on Evidence of Mediterranean Diet and Cardiovascular Disease: Are Individual Components Equal? Critical reviews in food science and nutrition 2015:0. doi: 10.1080/10408398.2015.1107021 | Other exposure |
| Mayhew AJ, de Souza RJ, Meyre D, et al. A systematic review and meta-analysis of nut consumption and incident risk of CVD and all-cause mortality. Br J Nutr 2016;115(2):212-25. doi: 10.1017/s0007114515004316 | Other exposure |
| Wu YH, Qian YF, Pan YW, et al. Association between dietary fiber intake and risk of coronary heart disease: A meta-analysis. Clinical Nutrition 2015;34(4):603-11. doi: 10.1016/j.clnu.2014.05.009 | Other exposure |
| Rizos EC, Ntzani EE, Bika E, et al. Association between omega-3 fatty acid supplementation and risk of major cardiovascular disease events: A systematic review and meta-analysis. JAMA - Journal of the American Medical Association 2012;308(10):1024-33 | Clinical population |
| Li F, Hou LN, Chen W, et al. Associations of dietary patterns with the risk of all-cause, CVD and stroke mortality: A meta-Analysis of prospective cohort studies. British Journal of Nutrition 2015;113(1):16-24. | Other exposure |
| Hunter JE, Zhang J, Kris-Etherton PM. Cardiovascular disease risk of dietary stearic acid compared with trans, other saturated, and unsaturated fatty acids: a systematic review. Am J Clin Nutr 2010;91(1):46-63. doi: 10.3945/ajcn.2009.27661 | Other outcome |
| Gan Y, Tong XY, Li LQ, et al. Consumption of fruit and vegetable and risk of coronary heart disease: A meta-analysis of prospective cohort studies. International Journal of Cardiology 2015;183:129-37. doi: 10.1016/j.ijcard.2015.01.077 | Other outcome |
| Brennan SF, Woodside JV, Lunny PM, et al. Dietary fat and breast cancer mortality: a systematic review and meta-analysis. Critical reviews in food science and nutrition 2015 doi: 10.1080/10408398.2012.724481 | Other outcome |
| Kim Y, Je Y. Dietary fiber intake and total mortality: A meta-analysis of prospective cohort studies. American journal of epidemiology 2014;180(6):565-73. | Other exposure |
| Hartley L, May MD, Loveman E, et al. Dietary fibre for the primary prevention of cardiovascular disease. Cochrane Database of Systematic Reviews 2016; (1). http://onlinelibrary.wiley.com/doi/10.1002/14651858.CD011472.pub2/abstract. | Other exposure |
| Zhang XY, Shu L, Si CJ, et al. Dietary Patterns, Alcohol Consumption and Risk of Coronary Heart Disease in Adults: A Meta-Analysis. Nutrients 2015;7(8):6582-605. doi: 10.3390/nu7085300 | Other exposure |
| Marik PE, Flemmer M. Do Dietary Supplements Have Beneficial Health Effects in Industrialized Nations: What Is the Evidence? Journal of Parenteral and Enteral Nutrition 2012;36(2):159-68. doi: 10.1177/0148607111416485 | Not systematic review |
| Sonestedt E, Overby NC, Laaksonen DE, et al. Does high sugar consumption exacerbate cardiometabolic risk factors and increase the risk of type 2 diabetes and cardiovascular disease? Food & nutrition research 2012;56 doi: 10.3402/fnr.v56i0.19104 | Not systematic review |
| Aburto NJ, Hanson S, Gutierrez H, et al. Effect of increased potassium intake on cardiovascular risk factors and disease: systematic review and meta-analyses. Bmj-British Medical Journal 2013;346 doi: 10.1136/bmj.f1378 | Other exposure |
| Mozaffarian D, Micha R, Wallace S. Effects on coronary heart disease of increasing polyunsaturated fat in place of saturated fat: a systematic review and meta-analysis of randomized controlled trials. PLoS medicine 2010;7(3):e1000252. doi: 10.1371/journal.pmed.1000252 | Clinical population |
| Bloomfield HE, Koeller E, Greer N, et al. Effects on Health Outcomes of a Mediterranean Diet With No Restriction on Fat Intake: A Systematic Review and Meta-analysis. Ann Intern Med 2016;165(7):491-500. doi: 10.7326/m16-0361 | Other exposure |
| Shin JY, Xun P, Nakamura Y, et al. Egg consumption in relation to risk of cardiovascular disease and diabetes: a systematic review and meta-analysis. American Journal of Clinical Nutrition 2013;98(1):146-59. doi: 10.3945/ajcn.112.051318 | Other exposure |
| Zhao LG, Sun JW, Yang Y, et al. Fish consumption and all-cause mortality: a meta-analysis of cohort studies. European Journal of Clinical Nutrition 2016;70(2):155-61. doi: 10.1038/ejcn.2015.72 | Other exposure |
| Aucoin M, Cooley K, Knee C, et al. Fish-Derived Omega-3 Fatty Acids and Prostate Cancer: A Systematic Review. Integrative cancer therapies 2016 doi: 10.1177/1534735416656052 | Other outcome |
| O'Sullivan TA, Hafekost K, Mitrou F, et al. Food sources of saturated fat and the association with mortality: a meta-analysis. American journal of public health 2013;103(9):e31-42. doi: 10.2105/ajph.2013.301492 | Other exposure |
| Pedersen AN, Kondrup J, Borsheim E. Health effects of protein intake in healthy adults: a systematic literature review. Food & nutrition research 2013;57 doi: 10.3402/fnr.v57i0.21245 | Other exposure |
| Wang Q, Afshin A, Yakoob MY, et al. Impact of Nonoptimal Intakes of Saturated, Polyunsaturated, and Trans Fat on Global Burdens of Coronary Heart Disease. Journal of the American Heart Association 2016;5(1) doi: 10.1161/jaha.115.002891 | Not systematic review |
| Hartley L, Igbinedion E, Holmes J, et al. Increased consumption of fruit and vegetables for the primary prevention of cardiovascular diseases. Cochrane Database Syst Rev 2013;6:Cd009874. doi: 10.1002/14651858.CD009874.pub2 | Other outcome |
| Pimpin L, Wu JHY, Haskelberg H, et al. Is butter back? A systematic review and meta-analysis of butter consumption and risk of cardiovascular disease, diabetes, and total mortality. PLoS ONE 2016;11(6) | Other exposure |
| Delgado-Lista J, Perez-Martinez P, Lopez-Miranda J, et al. Long chain omega-3 fatty acids and cardiovascular disease: A systematic review. British Journal of Nutrition 2012;107(SUPPL. 2):S201-S13. | Clinical population |
| D'Alessandro A, De Pergola G, Silvestris F. Mediterranean Diet and cancer risk: an open issue. International Journal of Food Sciences and Nutrition 2016;67(6):593-605. doi: 10.1080/09637486.2016.1191444 | Other outcome |
| Rees K, Hartley L, Flowers N, et al. 'Mediterranean' dietary pattern for the primary prevention of cardiovascular disease. Cochrane Database Syst Rev 2013;8:Cd009825. doi: 10.1002/14651858.CD009825.pub2 | Other exposure |
| Soedamah-Muthu SS, Ding EL, Al-Delaimy WK, et al. Milk and dairy consumption and incidence of cardiovascular diseases and all-cause mortality: dose-response meta-analysis of prospective cohort studies. Am J Clin Nutr 2011;93(1):158-71. doi: 10.3945/ajcn.2010.29866 | Other exposure |
| Ramsden CE, Hibbeln JR, Majchrzak SF, et al. N-6 Fatty acid-specific and mixed polyunsaturate dietary interventions have different effects on CHD risk: A meta-analysis of randomised controlled trials. British Journal of Nutrition 2010;104(11):1586-600. | Clinical population |
| Luo C, Zhang Y, Ding Y, et al. Nut consumption and risk of type 2 diabetes, cardiovascular disease, and all-cause mortality: a systematic review and meta-analysis. Am J Clin Nutr 2014;100(1):256-69. doi: 10.3945/ajcn.113.076109 | Other exposure |
| Grosso G, Yang J, Marventano S, et al. Nut consumption on all-cause, cardiovascular, and cancer mortality risk: A systematic review and meta-analysis of epidemiologic studies. American Journal of Clinical Nutrition 2015;101(4):783-93. | Other exposure |
| Kotwal S, Jun M, Sullivan D, et al. Omega 3 fatty acids and cardiovascular outcomes: Systematic review and meta-analysis. Circulation: Cardiovascular Quality and Outcomes 2012;5(6):808-18. | Clinical population |
| Al-Khudairy L, Hartley L, Clar C, et al. Omega 6 fatty acids for the primary prevention of cardiovascular disease. Cochrane Database of Systematic Reviews 2015; (11). http://onlinelibrary.wiley.com/doi/10.1002/14651858.CD011094.pub2/abstract. | Other outcome |
| Kruse LG, Ogletree RL, Jr. Omega-3 fatty acids and cardiovascular risk. Journal of the Mississippi State Medical Association 2013;54(6):156-7. | Clinical population |
| Price HC, Simmons RK. Primary prevention of CVD: diet. BMJ clinical evidence 2011;2011 | Not systematic review |
| Larsson SC, Orsini N. Red Meat and Processed Meat Consumption and All-Cause Mortality: A Meta-Analysis. American journal of epidemiology 2014;179(3):282-89. doi: 10.1093/aje/kwt261 | Other exposure |
| Adler AJ, Taylor F, Martin N, et al. Reduced dietary salt for the prevention of cardiovascular disease. Cochrane Database Syst Rev 2014;12:Cd009217. doi: 10.1002/14651858.CD009217.pub3 | Clinical population |
| Hooper L, Summerbell CD, Thompson R, et al. Reduced or modified dietary fat for preventing cardiovascular disease. Cochrane Database of Systematic Reviews 2012(5) doi: 10.1002/14651858.CD002137.pub3 | Clinical population |
| Hooper L, Martin N, Abdelhamid A, et al. Reduction in saturated fat intake for cardiovascular disease. Cochrane Database Syst Rev 2015(6):Cd011737. doi: 10.1002/14651858.cd011737 | Clinical population |
| van den Brandt PA, Schouten LJ. Relationship of tree nut, peanut and peanut butter intake with total and cause-specific mortality: a cohort study and meta-analysis. Int J Epidemiol 2015;44(3):1038-49. doi: 10.1093/ije/dyv039 | Other exposure |
| Lorente-Cebrian S, Costa AG, Navas-Carretero S, et al. Role of omega-3 fatty acids in obesity, metabolic syndrome, and cardiovascular diseases: a review of the evidence. Journal of physiology and biochemistry 2013;69(3):633-51. doi: 10.1007/s13105-013-0265-4 | Not systematic review |
| Engell RE, Sanman E, Lim SS, et al. Seafood omega-3 intake and risk of coronary heart disease death: an updated meta-analysis with implications for attributable burden. Lancet (London, England) 2013;381:45-45. | Abstract only |
| Sethi A, Bajaj A, Khosla S, et al. Statin use mitigate the benefit of omega-3 fatty acids supplementation - A meta-regression of randomized trials. American Journal of Therapeutics 2016;23(3):e737-e48. | Other exposure |
| Zhang C, Qin YY, Wei X, et al. Tea consumption and risk of cardiovascular outcomes and total mortality: a systematic review and meta-analysis of prospective observational studies. European Journal of Epidemiology 2015;30(2):103-13. doi: 10.1007/s10654-014-9960-x | Other exposure |
| Elwood PC, Pickering JE, Givens DI, et al. The Consumption of Milk and Dairy Foods and the Incidence of Vascular Disease and Diabetes: An Overview of the Evidence. Lipids 2010;45(10):925-39. doi: 10.1007/s11745-010-3412-5 | Other exposure |
| The role of Mediterranean type of diet on the development of cancer and cardiovascular disease, in the elderly: a systematic review | Other exposure |
| Johnson C, Raj TS, Trieu K, et al. The Science of Salt: A Systematic Review of Quality Clinical Salt Outcome Studies June 2014 to May 2015. Journal of Clinical Hypertension 2016;18(9):832-39. | Other outcome |
| Lippi G, Mattiuzzi C, Franchini M. Vegetables intake and venous thromboembolism: a systematic review. Blood Coagulation & Fibrinolysis 2016;27(3):242-45. doi: 10.1097/mbc.0000000000000427 | Other outcome |
| Elamin MB, Abu Elnour NO, Elamin KB, et al. Vitamin D and cardiovascular outcomes: A systematic review and meta-analysis. Journal of Clinical Endocrinology and Metabolism 2011;96(7):1931-42. | Other exposure |
| Wei HL, Gao Z, Liang R, et al. Whole-grain consumption and the risk of all-cause, CVD and cancer mortality: a meta-analysis of prospective cohort studies. British Journal of Nutrition 2016;116(3):514-25. doi: 10.1017/s0007114516001975 | Other exposure |
| ***Statin*** |  |
| Alberton M, Wu P, Druyts E, et al. Adverse events associated with individual statin treatments for cardiovascular disease: an indirect comparison meta-analysis. QJM : monthly journal of the Association of Physicians 2012;105(2):145-57. doi: 10.1093/qjmed/hcr158 | Other outcome |
| Silverman MG, Ference BA, Im K, et al. Association Between Lowering LDL-C and Cardiovascular Risk Reduction Among Different Therapeutic Interventions: A Systematic Review and Meta-analysis. Jama 2016;316(12):1289-97. doi: 10.1001/jama.2016.13985 | Data Extraction: did not provide data from individual studies or did not perform meta-analysis |
| Danaei G, Tavakkoli M, Hernan MA. Bias in Observational Studies of Prevalent Users: Lessons for Comparative Effectiveness Research From a Meta-Analysis of Statins. American journal of epidemiology 2012;175(4):250-62. doi: 10.1093/aje/kwr301 | Clinical population |
| Hourcade-Potelleret F, Laporte S, Lehnert V, et al. Clinical benefit from pharmacological elevation of high-density lipoprotein cholesterol: meta-regression analysis. Heart (British Cardiac Society) 2015;101(11):847-53. doi: 10.1136/heartjnl-2014-306691 | Clinical population |
| Naci H, Brugts JJ, Fleurence R, et al. Comparative benefits of statins in the primary and secondary prevention of major coronary events and all-cause mortality: a network meta-analysis of placebo-controlled and active-comparator trials. European journal of preventive cardiology 2013;20(4):641-57. doi: 10.1177/2047487313480435 | Data Extraction: did not provide data from individual studies or did not perform meta-analysis |
| Cifkova R, Krajcoviechova A. Dyslipidemia and Cardiovascular Disease in Women. Current Cardiology Reports 2015;17(7) | Not systematic review |
| Thomopoulos C, Skalis G, Michalopoulou H, et al. Effect of low-density lipoprotein cholesterol lowering by ezetimibe/simvastatin on outcome incidence: Overview, meta-analyses, and meta-regression analyses of randomized trials. Clinical Cardiology 2015;38(12):763-69. | Other outcome |
| Fulcher J, O'Connell R, Voysey M, et al. Efficacy and safety of LDL-lowering therapy among men and women: Meta-analysis of individual data from 174 000 participants in 27 randomised trials. The Lancet 2015;385(9976):1397-405. | Not systematic review |
| Lu Y, Cheng Z, Zhao Y, et al. Efficacy and safety of long-term treatment with statins for coronary heart disease: A Bayesian network meta-analysis. Atherosclerosis 2016;254:215-27. | Data Extraction: did not provide data from individual studies or did not perform meta-analysis |
| Baigent C, Blackwell L, Emberson J, et al. Efficacy and safety of more intensive lowering of LDL cholesterol: a meta-analysis of data from 170,000 participants in 26 randomised trials. Lancet (London, England) 2010;376(9753):1670-81. doi: 10.1016/s0140-6736(10)61350-5 | Not systematic review |
| Koduri H, Kanmanthareddy A, Akinapelli A, et al. Efficacy of ezetimibe and statin versus statin: A meta-analysis of randomized controlled trials. Journal of the American College of Cardiology 2016;67(13 SUPPL. 1):1884. | Abstract only |
| Bruckert E, Ferrieres J. Evidence supporting primary prevention of cardiovascular diseases with statins: Gaps between updated clinical results and actual practice. Archives of cardiovascular diseases 2014;107(3):188-200. doi: 10.1016/j.acvd.2014.01.011 | Not systematic review |
| Takagi H, Matsui M, Umemoto T. High-density lipoprotein-dependent effects of statins on the risk of coronary heart disease deaths and events. International Journal of Cardiology 2011;152(3):377-79. doi: 10.1016/j.ijcard.2011.07.071 | Not systematic review |
| Ribeiro RA, Ziegelmann PK, Duncan BB, et al. Impact of statin dose on major cardiovascular events: a mixed treatment comparison meta-analysis involving more than 175,000 patients. Int J Cardiol 2013;166(2):431-9. doi: 10.1016/j.ijcard.2011.10.128 | Data Extraction: did not provide data from individual studies or did not perform meta-analysis |
| Mills EJ, O'Regan C, Eyawo O, et al. Intensive statin therapy compared with moderate dosing for prevention of cardiovascular events: a meta-analysis of >40 000 patients. Eur Heart J 2011;32(11):1409-15. doi: 10.1093/eurheartj/ehr035 | Clinical population |
| Chan DKY, O'Rourke F, Shen Q, et al. Meta-analysis of the cardiovascular benefits of intensive lipid lowering with statins. Acta Neurologica Scandinavica 2011;124(3):188-95. doi: 10.1111/j.1600-0404.2010.01450.x | Other outcome |
| Liu G, Zheng XX, Xu YL, et al. Meta-analysis of the effect of statins on mortality in patients with preserved ejection fraction. American Journal of Cardiology 2014;113(7):1198-204. | Clinical population |
| Hansen MR, Pottegard A, Hrobjartsson A, et al. Modelling of endpoint postponement for all-cause mortality in statin trials. Pharmacoepidemiology and Drug Safety 2015;24:1. | Abstract only |
| Karlson BW, Palmer MK, Nicholls SJ, et al. Predicting the reduction in risk of cardiovascular events with high-intensity statin treatment: A voyager analysis. Atherosclerosis 2015;241(1):e202-e03. | Abstract only |
| Sethi A, Bajaj A, Khosla S, et al. Statin use mitigate the benefit of omega-3 fatty acids supplementation - A meta-regression of randomized trials. American Journal of Therapeutics 2016;23(3):e737-e48. | Clinical population |
| Beri A, Contractor T, Khasnis A, et al. Statins and the reduction of sudden cardiac death: Antiarrhythmic or anti-ischemic effect? American Journal of Cardiovascular Drugs 2010;10(3):155-64. | Not systematic review |
| Squizzato A, Romualdi E, Dentali F, et al. Statins for acute ischemic stroke. Cochrane database of systematic reviews (Online) 2011(8):CD007551. | Clinical population |
| Minder CM, Blumenthal RS, Blaha MJ. Statins for primary prevention of cardiovascular disease: The benefits outweigh the risks. Current Opinion in Cardiology 2013;28(5):554-60. | Not systematic review |
| Mihaylova B, Emberson J, Blackwell L, et al. The effects of lowering LDL cholesterol with statin therapy in people at low risk of vascular disease: meta-analysis of individual data from 27 randomised trials. Lancet (London, England) 2012;380(9841):581-90. doi: 10.1016/s0140-6736(12)60367-5 | Data Extraction: did not provide data from individual studies or did not perform meta-analysis |
| Briel M, Vale N, Schwartz GG, et al. Updated evidence on early statin therapy for acute coronary syndromes: meta-analysis of 18 randomized trials involving over 14,000 patients. Int J Cardiol 2012;158(1):93-100. doi: 10.1016/j.ijcard.2011.01.033 | Clinical population |
