# Supplementary material for: Reporting bias in the literature on the associations of health-related behaviors and statins with cardiovascular disease and all-cause mortality
Source: PLoS Biol. 2018 Jun 18;16(6):e2005761. doi: 10.1371/journal.pbio.2005761 (PMC6023226; doi:10.1371/journal.pbio.2005761)
Supplement: S2 Table — (DOC) [file pbio.2005761.s004.doc]

**S2 Table: Summary of reasons for excluding studies during full-text screening, by research area.**

|  | Did not include the exposure of interest | Did not include cardiovascular disease mortality or all-cause mortality outcomes | Did not provide data from individual studies or did not perform meta-analysis | Did not published full-text  (abstract only) | Chinese language | Clinical population | It was not a systematic review of literature |
| --- | --- | --- | --- | --- | --- | --- | --- |
| Physical activity | 41% | 29% | 18% | - | - | - | 12% |
| Sedentary Behavior | 11% | 11% | 56% | - | - | - | 22% |
| Alcohol | 12% | 27% | 8% | 4% | 8% | 23% | 19% |
| Smoking | 22% | 11% | 33% | 11% | - | - | 22% |
| Diet | 49% | 17% | 6% | 2% | - | 17% | 9% |
| Statins | - | 12% | 20% | 12% | - | 28% | 28% |
| Overall | 28% | 19% | 15% | 4% | 1% | 16% | 17% |
