# Supplementary material for: Reporting bias in the literature on the associations of health-related behaviors and statins with cardiovascular disease and all-cause mortality
Source: PLoS Biol. 2018 Jun 18;16(6):e2005761. doi: 10.1371/journal.pbio.2005761 (PMC6023226; doi:10.1371/journal.pbio.2005761)
Supplement: S3 Table — (DOC) [file pbio.2005761.s005.doc]

**S3 Table:** Risk of bias in systematic reviews of physical activity, sedentary behaviour, diet, alcohol, smoking and statins using ROBIS tool.

|  | **DOMAIN 1:**  **STUDY ELIGIBILITY** | | | | |  | **DOMAIN 2:**  **IDENTIFICATION AND SELECTION** | | | | |  | **DOMAIN 3:**  **DATA COLLECTION AND STUDY APPRAISAL** | | | | |  | **DOMAIN 4:**  **SYNTHESIS AND FINDINGS** | | | | | |  |
| --- | --- | --- | --- | --- | --- | --- | --- | --- | --- | --- | --- | --- | --- | --- | --- | --- | --- | --- | --- | --- | --- | --- | --- | --- | --- |
| Study | 1.1 | 1.2 | 1.3 | 1.4 | 1.5 | **Concerns eligibility criteria** | 2.1 | 2.2 | 2.3 | 2.4 | 2.5 | **Concerns selection studies** | 3.1 | 3.2 | 3.3 | 3.4 | 3.5 | **Concerns collection of data** | 4.1 | 4.2 | 4.3 | 4.4 | 4.5 | 4.6 | **Concerns synthesis** |
| ***Physical activity*** |  |  |  |  |  |  |  |  |  |  |  |  |  |  |  |  |  |  |  |  |  |  |  |  |  |
| Kelly, 2014[1] | Y | PY | Y | Y | PY | LOW | Y | Y | Y | N | Y | HIGH | N | Y | Y | Y | Y | HIGH | Y | Y | PN | Y | PY | Y | HIGH |
| Samitz, 2011[2] | PY | Y | Y | Y | Y | LOW | Y | Y | Y | Y | PY | LOW | Y | Y | Y | N | N | HIGH | Y | NI | Y | Y | PY | N | HIGH |
| Woodcock, 2011[3] | PY | Y | Y | PN | Y | HIGH | Y | Y | N | Y | PY | HIGH | NI | PY | Y | Y | NI | UNCLEAR | Y | NI | Y | Y | Y | Y | LOW |
| Hupin, 2015[4] | PY | Y | Y | PY | PY | LOW | Y | Y | PN | N | PY | HIGH | NI | Y | Y | Y | NI | UNCLEAR | Y | NI | Y | Y | Y | PY | LOW |
| ***Sedentary behaviour*** |  |  |  |  |  |  |  |  |  |  |  |  |  |  |  |  |  |  |  |  |  |  |  |  |  |
| Biswas, 2015[5] | PY | Y | Y | Y | PY | LOW | Y | Y | Y | N | PY | HIGH | PY | Y | Y | Y | Y | LOW | Y | NI | N | Y | N | N | HIGH |
| Chau, 2013[6] | PY | Y | Y | PN | PY | HIGH | Y | Y | PN | N | NI | HIGH | PY | Y | Y | PY | Y | LOW | Y | NI | Y | Y | N | Y | HIGH |
| Grontved, 2011[7] | PY | PY | PY | Y | Y | LOW | Y | Y | PY | N | NI | HIGH | PY | Y | Y | N | NI | HIGH | Y | NI | Y | Y | Y | N | HIGH |
| Wilmot, 2012[8] | PY | PN | PN | Y | PY | HIGH | Y | Y | N | N | Y | HIGH | PY | Y | Y | Y | Y | LOW | Y | NI | N | Y | PN | N | HIGH |
| Ford, 2012[9] | PY | PN | PN | Y | PY | HIGH | N | Y | Y | N | NI | HIGH | Y | Y | PN | N | N | HIGH | NI | NI | N | Y | N | N | HIGH |
| Pandey, 2016[10] | PY | PN | PN | Y | PY | HIGH | Y | Y | PN | N | Y | HIGH | Y | Y | Y | Y | NI | UNCLEAR | Y | NI | Y | Y | Y | Y | UNCLEAR |
| Sun, 2015[11] | PY | PN | PN | Y | NI | HIGH | Y | Y | Y | NI | NI | UNCLEAR | PY | Y | Y | N | NI | HIGH | Y | NI | Y | Y | Y | N | HIGH |
| ***Alcohol*** |  |  |  |  |  |  |  |  |  |  |  |  |  |  |  |  |  |  |  |  |  |  |  |  |  |
| Costanzo, 2011[12] | PY | PN | PN | PY | PY | HIGH | PY | Y | PN | N | Y | HIGH | NI | Y | Y | Y | NI | UNCLEAR | Y | NI | Y | Y | Y | PN | HIGH |
| Jayasekara, 2014[13] | PY | PN | PN | PY | PY | LOW | Y | N | Y | N | N | HIGH | PY | Y | Y | N | N | HIGH | Y | NI | Y | Y | Y | N | HIGH |
| Roerecke, 2011[14] | PY | Y | Y | Y | PY | LOW | Y | Y | Y | N | N | HIGH | NI | Y | Y | N | NI | HIGH | Y | NI | Y | Y | PY | N | LOW |
| Roerecke, 2014 [15] | Y | Y | Y | PY | PY | LOW | Y | Y | Y | N | N | HIGH | N | Y | Y | N | N | HIGH | Y | Y | Y | Y | PY | N | HIGH |
| Ronksley, 2011[16] | PY | PY | PY | Y | Y | LOW | Y | Y | Y | Y | Y | LOW | PY | PN | Y | PY | NI | HIGH | Y | PY | Y | Y | Y | PY | LOW |
| Park, 2015[17] | N | N | PN | NI | NI | HIGH | Y | Y | N | NI | PY | HIGH | NI | PN | PN | Y | Y | HIGH | Y | NI | PN | Y | PN | Y | HIGH |
| Stockwell, 2016[18] | Y | Y | Y | Y | PY | LOW | Y | Y | Y | N | PY | HIGH | Y | Y | Y | PY | NI | UNCLEAR | Y | Y | Y | Y | Y | Y | LOW |
| Zheng, 2015[19] | PY | PN | PN | PY | Y | HIGH | Y | Y | Y | Y | PY | LOW | Y | Y | Y | Y | Y | LOW | Y | NI | Y | Y | Y | Y | LOW |
| Roerecke, 2010[20] | PY | PN | PN | Y | PY | HIGH | Y | Y | Y | N | N | HIGH | NI | Y | Y | N | N | HIGH | Y | NI | Y | Y | PY | N | HIGH |
| Roerecke, 2014[21] | Y | Y | Y | Y | PY | LOW | Y | Y | Y | N | NI | HIGH | N | Y | Y | N | N | HIGH | Y | Y | Y | Y | Y | N | HIGH |
| ***Smoking*** |  |  |  |  |  |  |  |  |  |  |  |  |  |  |  |  |  |  |  |  |  |  |  |  |  |
| Gellert, 2012[22] | PY | Y | Y | Y | Y | LOW | Y | Y | PN | NI | PY | UNCLEAR | PY | Y | Y | Y | NI | UNCLEAR | Y | NI | Y | Y | Y | Y | UNCLEAR |
| Lv, 2015[23] | PY | Y | Y | Y | PY | LOW | Y | Y | Y | N | Y | HIGH | Y | PN | Y | Y | NI | UNCLEAR | Y | NI | Y | PY | Y | N | HIGH |
| Sinha, 2016[24] | PY | Y | Y | PY | PY | LOW | Y | Y | Y | N | PY | HIGH | PY | PN | Y | N | Y | HIGH | Y | NI | PN | Y | PN | N | HIGH |
| ***Diet*** |  |  |  |  |  |  |  |  |  |  |  |  |  |  |  |  |  |  |  |  |  |  |  |  |  |
| Farvid, 2014[25] | PY | Y | PY | Y | Y | LOW | Y | Y | Y | Y | N | HIGH | Y | Y | PY | N | N | HIGH | Y | NI | PY | Y | Y | N | HIGH |
| Graudal, 2014[26] | Y | Y | Y | Y | Y | LOW | Y | Y | PY | Y | NI | HIGH | Y | NI | Y | N | NI | HIGH | NI | NI | PY | PY | N | N | HIGH |
| Hu, 2014[27] | Y | Y | PY | PY | Y | LOW | Y | Y | N | Y | NI | HIGH | NI | Y | Y | Y | NI | HIGH | Y | NI | PY | PY | PY | N | HIGH |
| Li, 2012[28] | Y | PY | N | PN | PN | HIGH | Y | Y | PN | N | N | HIGH | Y | Y | Y | N | N | HIGH | Y | NI | N | Y | PY | N | HIGH |
| Musa-Veloso, 2011[29] | Y | PY | PY | Y | N | HIGH | Y | PY | PY | PY | NI | HIGH | Y | Y | PY | N | N | HIGH | Y | NI | Y | N | N | N | HIGH |
| Pan, 2012[30] | Y | Y | Y | Y | N | HIGH | Y | Y | Y | N | N | HIGH | Y | Y | Y | Y | Y | LOW | Y | NI | N | PY | PY | N | HIGH |
| Poggio, 2015[31] | Y | Y | PY | Y | Y | LOW | Y | Y | N | Y | Y | HIGH | NI | Y | Y | Y | Y | HIGH | Y | NI | Y | Y | Y | Y | LOW |
| Schwingshackl, 2014[32] | Y | PY | PY | Y | Y | LOW | Y | Y | PY | Y | PY | LOW | NI | Y | Y | Y | N | HIGH | PY | NI | Y | Y | PN | Y | HIGH |
| Wang, 2014[33] | Y | Y | Y | Y | Y | LOW | Y | Y | N | Y | Y | HIGH | Y | Y | Y | Y | PY | LOW | Y | NI | Y | Y | Y | Y | HIGH |
| Chen, 2016[34] | Y | Y | Y | Y | Y | LOW | Y | Y | PY | Y | Y | LOW | Y | Y | PY | Y | Y | LOW | Y | NI | Y | Y | Y | Y | LOW |
| Cheng, 2015[35] | Y | Y | Y | Y | Y | LOW | Y | Y | PY | Y | N | HIGH | Y | Y | Y | Y | Y | LOW | Y | NI | Y | Y | Y | NI | HIGH |
| Cheng, 2016[36] | Y | Y | Y | Y | Y | LOW | Y | Y | PN | Y | N | HIGH | Y | Y | Y | Y | Y | LOW | Y | NI | Y | Y | Y | N | HIGH |
| De Souza, 2015[37] | Y | Y | Y | Y | Y | LOW | Y | Y | Y | Y | N | HIGH | Y | Y | PY | Y | N | HIGH | PY | NI | Y | Y | Y | Y | LOW |
| Narain, 2016[38] | Y | Y | Y | Y | Y | LOW | Y | Y | PY | Y | Y | LOW | Y | Y | Y | N | Y | HIGH | Y | NI | Y | Y | Y | Y | LOW |
| ***Statins*** |  |  |  |  |  |  |  |  |  |  |  |  |  |  |  |  |  |  |  |  |  |  |  |  |  |
| Bukkapatnam, 2010[39] | Y | Y | Y | PN | N | HIGH | Y | N | PN | N | N | HIGH | Y | Y | Y | N | N | HIGH | Y | NI | Y | Y | Y | N | HIGH |
| Kizer, 2010[40] | Y | Y | Y | PY | Y | LOW | Y | Y | PN | N | N | HIGH | N | Y | N | N | N | HIGH | N | NI | Y | PY | PY | N | HIGH |
| Kostis, 2012[41] | Y | Y | Y | N | Y | HIGH | Y | N | Y | Y | Y | HIGH | N | Y | NI | Y | N | HIGH | Y | NI | Y | PY | PY | Y | LOW |
| Lv, 2014[42] | Y | PN | Y | PY | Y | HIGH | Y | Y | PN | Y | N | HIGH | Y | Y | PY | Y | N | HIGH | Y | NI | PY | Y | Y | PY | LOW |
| Ray, 2010[43] | PN | PN | PN | Y | Y | HIGH | Y | Y | N | PY | N | HIGH | Y | Y | Y | N | N | HIGH | Y | NI | Y | Y | Y | N | HIGH |
| Savarese, 2013[44] | Y | Y | Y | Y | Y | LOW | Y | N | Y | Y | Y | HIGH | Y | Y | Y | Y | Y | LOW | Y | NI | Y | Y | Y | Y | LOW |
| Taylor, 2011[45] | Y | Y | PY | Y | Y | LOW | Y | Y | Y | Y | Y | LOW | Y | Y | Y | Y | Y | LOW | Y | NI | Y | Y | Y | Y | LOW |
| Tonelli, 2011[46] | Y | Y | Y | Y | Y | LOW | Y | Y | Y | N | Y | HIGH | Y | Y | Y | Y | Y | LOW | Y | NI | Y | Y | Y | PN | HIGH |
| Chou, 2016[47] | Y | Y | Y | Y | Y | LOW | Y | Y | Y | N | Y | HIGH | Y | Y | PY | Y | Y | LOW | Y | NI | Y | Y | Y | PY | LOW |
| Preiss, 2015[48] | Y | Y | Y | PY | Y | LOW | Y | N | PN | N | Y | HIGH | Y | Y | Y | N | N | HIGH | PY | NI | Y | Y | Y | PY | LOW |
| Teng, 2015[49] | Y | Y | Y | Y | Y | LOW | Y | Y | Y | N | Y | HIGH | Y | Y | PY | Y | Y | LOW | Y | NI | Y | Y | PY | PY | LOW |
| Y=YES, PY= PROBABLY YES, PN=PROBABLY NO, N=NO, NI=NO INFORMATION | | | | | | | | | | | | | | | | | | | | | | | | |  |

**ROBIS: Tool to assess risk of bias in systematic reviews**

Phase 2: Identifying concerns with the review process

| **DOMAIN 1: STUDY ELIGIBILITY CRITERIA** | |
| --- | --- |
| Describe the study eligibility criteria, any restrictions on eligibility and whether there was evidence that objectives and eligibility criteria were pre-specified: | |
| 1.1 Did the review adhere to pre-defined objectives and eligibility criteria? | Y/PY/PN/N/NI |
| 1.2 Were the eligibility criteria appropriate for the review question? | Y/PY/PN/N/NI |
| 1.3 Were eligibility criteria unambiguous? | Y/PY/PN/N/NI |
| 1.4 Were all restrictions in eligibility criteria based on study characteristics  appropriate (e.g. date, sample size, study quality, outcomes measured)? | Y/PY/PN/N/NI |
| 1.5 Were any restrictions in eligibility criteria based on sources of  information appropriate (e.g. publication status or format, language, availability of data)? | Y/PY/PN/N/NI |
| Concerns regarding specification of study eligibility criteria | LOW/HIGH/UNCLEAR |
| Rationale for concern: |  |

| **DOMAIN 2: IDENTIFICATION AND SELECTION OF STUDIES** | |
| --- | --- |
| Describe methods of study identification and selection (e.g. number of reviewers involved): | |
| 2.1 Did the search include an appropriate range of databases/electronic sources for published and unpublished reports? | Y/PY/PN/N/NI |
| 2.2 Were methods additional to database searching used to identify  relevant reports? | Y/PY/PN/N/NI |
| 2.3 Were the terms and structure of the search strategy likely to retrieve  as many eligible studies as possible? | Y/PY/PN/N/NI |
| 2.4 Were restrictions based on date, publication format, or language  appropriate? | Y/PY/PN/N/NI |
| 2.5 Were efforts made to minimise error in selection of studies? | Y/PY/PN/N/NI |
| Concerns regarding methods used to identify and/or select studies | LOW/HIGH/UNCLEAR |
| Rationale for concern: |  |

| **DOMAIN 3: DATA COLLECTION AND STUDY APPRAISAL** | |
| --- | --- |
| Describe methods of data collection, what data were extracted from studies or collected through other means, how risk of bias was assessed (e.g. number of reviewers involved) and the tool used to assess risk of bias: | |
| 3.1 Were efforts made to minimise error in data collection? | Y/PY/PN/N/NI |
| 3.2 Were sufficient study characteristics available for both review authors  and readers to be able to interpret the results? | Y/PY/PN/N/NI |
| 3.3 Were all relevant study results collected for use in the synthesis? | Y/PY/PN/N/NI |
| 3.4 Was risk of bias (or methodological quality) formally assessed using  appropriate criteria? | Y/PY/PN/N/NI |
| 3.5 Were efforts made to minimise error in risk of bias assessment? | Y/PY/PN/N/NI |
| Concerns regarding methods used to collect data and appraise studies | LOW/HIGH/UNCLEAR |
| Rationale for concern: |  |

| **DOMAIN 4: SYNTHESIS AND FINDINGS** | |
| --- | --- |
| Describe synthesis methods: | |
| 4.1 Did the synthesis include all studies that it should? | Y/PY/PN/N/NI |
| 4.2 Were all pre-defined analyses reported or departures explained? | Y/PY/PN/N/NI |
| 4.3 Was the synthesis appropriate given the nature and similarity in  the research questions, study designs and outcomes across included studies? | Y/PY/PN/N/NI |
| 4.4 Was between-study variation (heterogeneity) minimal or  addressed in the synthesis? | Y/PY/PN/N/NI |
| 4.5 Were the findings robust, e.g. as demonstrated through funnel  plot or sensitivity analyses? | Y/PY/PN/N/NI |
| 4.6 Were biases in primary studies minimal or addressed in the  synthesis? | Y/PY/PN/N/NI |
| Concerns regarding the synthesis and findings | LOW/HIGH/UNCLEAR |
| Rationale for concern: |  |

Y=YES, PY=PROBABLY YES, PN=PROBABLY NO, N=NO, NI=NO INFORMATION

# Phase 3: Judging risk of bias

Summarize the concerns identified during the Phase 2 assessment:

| **Domain** | **Concern** | **Rationale for concern** |
| --- | --- | --- |
| 1. Concerns regarding specification of study  eligibility criteria |  |  |
| 2. Concerns regarding methods used to  identify and/or select studies |  |  |
| 3. Concerns regarding used to collect data  and appraise studies |  |  |
| 4. Concerns regarding the synthesis and  findings |  |  |

| **RISK OF BIAS IN THE REVIEW** | |
| --- | --- |
| Describe whether conclusions were supported by the evidence: | |
| A. Did the interpretation of findings address all of the concerns identified in Domains 1 to 4? | Y/PY/PN/N/NI |
| B. Was the relevance of identified studies to the review's research  question appropriately considered? | Y/PY/PN/N/NI |
| C. Did the reviewers avoid emphasizing results on the basis of their  statistical significance? | Y/PY/PN/N/NI |
| Risk of bias in the review | RISK: LOW/HIGH/UNCLEAR |

Y=YES, PY=PROBABLY YES, PN=PROBABLY NO, N=NO, NI=NO INFORMATION
