# Supplementary material for: Reporting bias in the literature on the associations of health-related behaviors and statins with cardiovascular disease and all-cause mortality
Source: PLoS Biol. 2018 Jun 18;16(6):e2005761. doi: 10.1371/journal.pbio.2005761 (PMC6023226; doi:10.1371/journal.pbio.2005761)
Supplement: S4 Table — (DOC) [file pbio.2005761.s006.doc]

**S4 Table**. Meta-analyses of cardiovascular disease and all-cause mortality by research area.

| **First author,**  **year (Reference)** | **Exposure-outcome association** | **Number of Estimates** | **Sample size** | **Number of Cases** | **Largest study,**  **RR (95% CI)^*^** | **Random Effect, RR (95% CI)**** | **Random Effects, P^#^** | **I^2^ (95% CI)** | **Egger's P†** | **Excess Significance** | | |
| --- | --- | --- | --- | --- | --- | --- | --- | --- | --- | --- | --- | --- |
|  |  |  |  |  |  |  |  |  |  | O | E**^‡^** | P-value**^⁑^** |
| *Physical Activity* | |  |  |  |  |  |  |  |  |  |  |  |
| Kelly, 2014[1] | walking and all-cause mortality | 14 | 279231 | 21119 | 0.89(0.84,0.95) | 0.87 (0.8 ,0.95) | 2.79E-03 | 86.8 (79.6 ,90.6) | 0.32 | 3 | 6.71 | NP |
| Kelly, 2014[1] | cycling and all-cause mortality | 7 | 188539 | 20607 | 0.88(0.84,0.93) | 0.91 (0.88 ,0.94) | 1.27E-08 | 0 (0 ,58.5) | 0.58 | 2 | 5.42 | NP |
| Samitz, 2011[2] | total physical activity and all-cause mortality | 23 | 395382 | 34274 | 0.61(0.57,0.65) | 0.63 (0.56 ,0.69) | 2.86E-18 | 85.8 (80.1 ,89.2) | 0.09 | 18 | 20.21 | NP |
| Samitz, 2011[2] | leisure-time physical activity and all-cause mortality | 41 | 551110 | 61465 | 0.67(0.62,0.72) | 0.73 (0.69 ,0.77) | 1.44E-31 | 71.1 (59.3 ,78.2) | 0.03 | 30 | 37.63 | NP |
| Samitz, 2011[2] | leisure-time physical activity and all-cause mortality | 6 | 384672 | 28607 | 0.99(0.99,1.00) | 0.95 (0.93 ,0.98) | 4.67E-05 | 96.9 (95.6 ,97.6) | 0.21 | 5 | 0.39 | <0.01 |
| Samitz, 2011[2] | exercise and all-cause mortality | 8 | 396431 | 16481 | 0.95(0.92,0.97) | 0.91 (0.87 ,0.94) | 4.73E-07 | 83.2 (65.8 ,89.8) | 0.06 | 8 | 1.77 | <0.01 |
| Samitz, 2011[2] | walking and all-cause mortality | 10 | 148627 | 7044 | 0.99(0.98,1.00) | 0.97 (0.94 ,0.99) | 3.35E-03 | 78.1 (55.5 ,86.6) | <0.01 | 5 | 0.52 | <0.01 |
| Samitz, 2011[2] | physical activity for transportation and all-cause mortality | 6 | 203914 | 14253 | 0.99(0.98,1.00) | 0.97 (0.94 ,1) | 2.51E-02 | 76.2 (29.5 ,87.6) | 0.15 | 2 | 0.34 | 0.04 |
| Samitz, 2011[2] | routine activities of daily living and all-cause mortality | 4 | 99618 | 4161 | 0.98(0.98,0.99) | 0.96 (0.93 ,0.98) | 1.36E-03 | 94.7 (90.2 ,96.6) | 0.32 | 3 | 0.26 | <0.01 |
| Woodcock, 2011[3] | moderate non-vigorous physical activity and all-cause mortality | 22 | 975227 | 64970 | 1.01(0.97,1.06) | 0.8 (0.76 ,0.85) | 1.35E-13 | 88.8 (84.8 ,91.3) | 0.07 | 18 | 1.4 | <0.01 |
| Woodcock, 2011[3] | walking and all-cause mortality | 5 | 217042 | 11383 | 0.95(0.90,1.01) | 0.89 (0.82 ,0.96) | 1.58E-03 | 75.9 (12.7 ,88.3) | 0.70 | 3 | 1.25 | 0.10 |
| Hupin, 2015 [4] | high dose physical activity and all-cause mortality | 9 | 122417 | 18122 | 0.75(0.70,0.80) | 0.65 (0.61 ,0.7) | 2.45E-33 | 61.1 (0 ,79.5) | 0.16 | 9 | 8.31 | 1.00 |
| *Sedentary Behavior* | |  |  |  |  |  |  |  |  |  |  |  |
| Biswas, 2015[5] | sedentary time and all-cause mortality | 12 | 836491 | 15644 | 1.05(1.03,1.07) | 1.19 (1.11 ,1.27) | 3.86E-07 | 89.8 (84.5 ,92.7) | 0.02 | 11 | 1.86 | <0.01 |
| Biswas, 2015[5] | sedentary time and cvd mortality | 6 | 528440 | 4383 | 1.23(1.14,1.32) | 1.18 (1.11 ,1.26) | 4.05E-07 | 35.6 (0 ,73.5) | 0.16 | 5 | 3.57 | 0.41 |
| Biswas, 2015[5] | high physical activity, high sedentary time and all-cause mortality | 6 | 741588 | 14394 | 1.12(1.03,1.22) | 1.17 (1.01 ,1.36) | 3.36E-02 | 81.1 (51.1 ,89.6) | 0.96 | 5 | 3.92 | 0.67 |
| Biswas, 2015[5] | low physical activity, high sedentary time and all-cause mortality | 6 | 741588 | 14394 | 1.29(1.20,1.38) | 1.46 (1.29 ,1.65) | 1.18E-09 | 86.9 (71.7 ,92.2) | 0.50 | 6 | 5.15 | 1.00 |
| Chau, 2013[6] | sedentary time and all-cause mortality | 6 | 595186 | 29162 | 1.02(1.02,1.03) | 1.02 (1.01 ,1.03) | 8.59E-05 | 84.3 (63.1 ,91) | 0.24 | 5 | 0.69 | <0.01 |
| Grontved, 2011[7] | television viewing and all-cause mortality | 3 | 26509 | 1879 | 1.14(1.06,1.23) | 1.13 (1.07 ,1.18) | 4.75E-06 | 0 (0 ,72.9) | 0.61 | 2 | 1.15 | 0.56 |
| Wilmot, 2012[8] | sedentary time and all-cause mortality | 8 | 497211 | 44998 | 1.81(1.74,1.88) | 1.66 (1.5 ,1.83) | 1.08E-22 | 79.3 (53.7 ,87.9) | 0.38 | 8 | 8 | NP |
| Wilmot, 2012[8] | sedentary time and cvd mortality | 8 | 421921 | 13023 | 1.95(1.82,2.10) | 1.94 (1.66 ,2.26) | 2.78E-17 | 62.7 (0 ,80.8) | 0.82 | 6 | 7.92 | NP |
| Ford, 2012 [9] | sitting time and cvd mortality | 2 | 364035 | 11053 | 1.05(1.03,1.08) | 1.03 (0.99 ,1.08) | 1.36E-01 | 86 (0 ,0) | NA | 1 | 1.14 | NP |
| Ford, 2012 [9] | screen-time and cvd mortality | 4 | 270560 | 5521 | 1.17(1.13,1.21) | 1.17 (1.13 ,1.21) | 5.24E-21 | 0 (0 ,67.9) | 0.94 | 2 | 1.88 | 1.00 |
| Pandey, 2016[10] | sedentary time and cvd mortality | 5 | 579948 | 19723 | 1.23(1.14,1.32) | 1.17 (1.08 ,1.26) | 7.18E-05 | 52.6 (0 ,80.6) | 0.54 | 4 | 4.85 | NP |
| Sun, 2015[11] | television viewing and all-cause mortality | 10 | 536683 | 50456 | 1.61(1.47,1.76) | 1.33 (1.2 ,1.47) | 4.19E-08 | 66.7 (20.2 ,81.3) | 0.74 | 7 | 8.72 | NP |
| *Alcohol* |  |  |  |  |  |  |  |  |  |  |  |  |
| Costanzo, 2011[12] | wine intake and all-cause mortality | 5 | 56610 | 11905 | 1.81(1.5,2.18) | 1.16 (0.74 ,1.82) | 5.26E-01 | 87.6 (70.7 ,92.9) | 0.84 | 1 | 5 | NP |
| Costanzo, 2011[12] | wine intake and chd mortality | 2 | 60773 | 1335 | 1.08(0.61,1.91) | 0.92 (0.6 ,1.42) | 7.16E-01 | 0 (0 ,0) | NA | 0 | 0.38 | NP |
| Costanzo, 2011[12] | wine intake and cvd mortality | 3 | 41401 | 1113 | 0.67(0.45,1) | 0.72 (0.49 ,1.06) | 9.66E-02 | 0 (0 ,72.9) | 0.14 | 0 | 2.61 | NP |
| Costanzo, 2011[12] | beer intake and all-cause mortality | 2 | 15545 | 8338 | 1.15(1.04,1.28) | 1.16 (1.05 ,1.29) | 5.03E-03 | 0 (0 ,0) | NA | 1 | 1.2 | NP |
| Costanzo, 2011[12] | beer intake and cvd mortality | 2 | 39573 | 866 | 0.74(0.37,1.48) | 0.92 (0.54 ,1.57) | 7.63E-01 | 0 (0 ,0) | NA | 0 | 1.59 | NP |
| Costanzo, 2011[12] | spirits intake and all-cause mortality | 2 | 15517 | 8338 | 1.12(0.94,1.34) | 1.12 (0.94 ,1.32) | 1.95E-01 | 0 (0 ,0) | NA | 0 | 1.11 | NP |
| Costanzo, 2011[12] | spirits intake and cvd mortality | 2 | 39573 | 866 | 0.88(0.47,1.64) | 1.47 (0.51 ,4.19) | 4.74E-01 | 77.7 (0 ,0) | NA | 1 | 0.45 | 040 |
| Jayasekara, 2014[13] | alcohol intake of >=60 g/day and all-cause mortality | 3 | 23599 | 3040 | 2.44(1.93,3.08) | 1.52 (0.78 ,2.98) | 2.19E-01 | 89.5 (62.9 ,94.8) | 0.54 | 1 | 3 | NP |
| Roerecke, 2011[14] | alcohol intake and ischemic heart disease mortality in men | 14 | 8038 | 408 | 1.08(0.92,1.26) | 1.29 (1.15 ,1.44) | 8.71E-06 | 26.4 (0 ,60.5) | 0.23 | 0 | 0.28 | NP |
| Roerecke, 2011[14] | alcohol intake and ischemic heart disease mortality in women | 10 | 0 | 0 | 1.09(0.97,1.22) | 1.54 (1.17 ,2.03) | 1.90E-03 | 71.1 (34.5 ,83.3) | 0.07 | NA | NA | NP |
| Roerecke, 2014[15] | heavy drinkers and ischemic heart disease mortality | 2 | 80233 | 862 | 1.06(0.76,1.48) | 1.07 (0.78 ,1.48) | 6.61E-01 |  | NA | 0 | 0.2 | NP |
| Ronksley, 2011 [16] | alcohol consumption and cvd mortality | 22 | 1176623 | 15628 | 0.71(0.68,0.75) | 0.75 (0.7 ,0.8) | 1.23E-17 | 72.2 (55 ,80.9) | 0.72 | 14 | 17.37 | NP |
| Ronksley, 2011 [16] | alcohol consumption and chd mortality | 30 | 1924612 | 47245 | 0.82(0.79,0.86) | 0.75 (0.68 ,0.81) | 6.08E-11 | 87.9 (84.2 ,90.4) | 0.56 | 18 | 17.79 | 1.00 |
| Ronksley, 2011 [16] | alcohol consumption and stroke mortality | 10 | 723551 | 5171 | 0.76(0.67,0.87) | 1.07 (0.89 ,1.27) | 4.87E-01 | 73.1 (40.8 ,84.2) | 0.17 | 4 | 6.63 | NP |
| Ronksley, 2011 [16] | alcohol consumption and all-cause mortality | 31 | 844127 | 96807 | 0.81(0.76,0.86) | 0.87 (0.83 ,0.92) | 1.40E-07 | 68.9 (53.1 ,77.7) | 0.90 | 9 | 24.35 | NP |
| Park, 2015[17] | mild alcohol consumption and all-cause mortality | 5 | 964893 | 18359 | 0.89(0.86,0.92) | 0.76 (0.59 ,0.97) | 2.74E-02 | 85.5 (62.7 ,92) | 0.31 | 3 | 2.27 | 0.66 |
| Stockwell, 2016 [18] | low alcohol consumption and all-cause mortality | 81 | 3674042 | 320252 | 0.95(0.91,0.99) | 0.86 (0.83 ,0.88) | 4.71E-22 | 63 (52 ,70.4) | 0.04 | 35 | 18.48 | <0.01 |
| Zheng, 2015[19] | alcohol intake and all-cause mortality in men | 9 | 88239 | 5409 | 1.09(1.01,1.17) | 1 (0.81 ,1.22) | 9.63E-01 | 91.4 (86.4 ,93.9) | 0.66 | 4 | 2.38 | 0.26 |
| Zheng, 2015[19] | alcohol intake and all-cause mortality in women | 9 | 100150 | 4503 | 1.07(0.96,1.19) | 1.2 (0.99 ,1.46) | 6.01E-02 | 68.6 (21.5 ,82.7) | 0.56 | 3 | 1.93 | 0.41 |
| Zheng, 2015[19] | alcohol intake and cardiac death in men | 4 | 51386 | 6230 | 1.15(1.02,1.28) | 0.93 (0.7 ,1.23) | 6.09E-01 | 86.9 (61.6 ,93.1) | 0.74 | 2 | 1.38 | 0.61 |
| Zheng, 2015[19] | alcohol intake and cardiac death in women | 4 | 67977 | 8553 | 0.88(0.8,0.97) | 1.04 (0.74 ,1.46) | 8.24E-01 | 48.1 (0 ,81.3) | 0.53 | 1 | 1.37 | NP |
| Roerecke, 2010[20] | alcohol consumption and ischemic heart disease mortality | 5 | 595 | 115 | 1.4(1.15,1.72) | 1.63 (1.28 ,2.09) | 8.12E-05 | 25.4 (0 ,72.4) | 0.01 | 5 | 1.54 | <0.01 |
| Roerecke, 2014[21] | alcohol consumption and ischemic heart disease mortality - reference group lifetime abstainers | 4 | 34234 | 618 | 0.74(0.56,0.97) | 1.05 (0.76 ,1.46) | 7.70E-01 | 69 (0 ,87.1) | 0.25 | 2 | 1.89 | 1.00 |
| Roerecke, 2014[21] | alcohol consumption and ischemic heart disease mortality - reference group current abstainers | 7 | 39461 | 818 | 0.92(0.84,1) | 0.9 (0.72 ,1.12) | 3.46E-01 | 67.5 (0 ,83.5) | 0.59 | 1 | 0.55 | 0.43 |
| *Smoking* |  |  |  |  |  |  |  |  |  |  |  |  |
| Gellert, 2012[22] | current smokers and all-cause mortality | 16 | 1194293 | 191660 | 2.48(2.47,2.5) | 1.88 (1.61 ,2.18) | 3.55E-16 | 97.7 (97.3 ,98) | 0.02 | 16 | 16 | NP |
| Gellert, 2012[22] | former smokers and all-cause mortality | 19 | 1220691 | 213874 | 1.22(1.16,1.28) | 1.33 (1.26 ,1.4) | 3.28E-25 | 60.4 (16 ,76.5) | 0.61 | 11 | 10.79 | 1.00 |
| Lv, 2015[23] | second hand smoking and all-cause mortality | 11 | 939163 | 27228 | 1.1(1.03,1.18) | 1.18 (1.1 ,1.27) | 1.54E-05 | 69.7 (33.8 ,82.2) | 0.12 | 6 | 6.15 | NP |
| Lv, 2015[23] | second hand smoking and cardiovascular mortality | 16 | 1367710 | 16738 | 1.02(0.98,1.07) | 1.16 (1.08 ,1.24) | 2.66E-05 | 67.1 (37.9 ,79.2) | <0.01 | 7 | 2.91 | 0.02 |
| Sinha, 2016[24] | smokeless tobacco use and all-cause mortality | 12 | 1099896 | 87321 | 0.89(0.84,0.93) | 1.22 (1.09 ,1.37) | 5.44E-04 | 95.3 (93.8 ,96.3) | 0.75 | 10 | 10.42 | NP |
| Sinha, 2016[24] | smokeless tobacco use and ischemic heart disease mortality | 13 | 909495 | 17746 | 1(0.92,1.08) | 1.1 (1.05 ,1.15) | 1.06E-05 | 8.1 (0 ,52.6) | 0.18 | 4 | 3.5 | 0.76 |
| Sinha, 2016[24] | smokeless tobacco use and stroke mortality | 8 | 873754 | 6123 | 1.46(1.31,1.64) | 1.39 (1.29 ,1.5) | 2.29E-18 | 0 (0 ,56.3) | 0.07 | 4 | 7.44 | NP |
| *Diet* |  |  |  |  |  |  |  |  |  |  |  |  |
| Farvid, 2014[25] | dietary linoleic acid and chd mortality | 11 | 270280 | 5882 | 0.77(0.63,0.94) | 0.79 (0.71 ,0.88) | 4.51E-05 | 0 (0 ,51.2) | 0.29 | 3 | 6.42 | NP |
| Graudal, 2014[26] | high sodium and all-cause mortality | 8 | 37618 | 8573 | 1(0.94,1.06) | 0.94 (0.87 ,1.03) | 1.76E-01 | 53.7 (0 ,77.3) | 0.91 | 3 | 0.4 | 0.01 |
| Hu, 2014[27] | fruits and vegetables and stroke mortality | 6 | 388003 | 4615 | 0.76(0.69,0.83) | 0.74 (0.62 ,0.88) | 5.82E-04 | 34.1 (0 ,73) | 0.43 | 3 | 4.83 | NP |
| Li, 2012[28] | salt intake and stroke mortality | 5 | 183532 | 4086 | 1.22(1.05,1.4) | 1.28 (1.1 ,1.5) | 1.44E-03 | 57.9 (0 ,82.3) | 0.24 | 3 | 3.38 | NP |
| Li, 2012[28] | salt intake and isquemic stroke mortality | 2 | 72085 | 647 | 2.04(1.41,2.94) | 2.16 (1.53 ,3.05) | 1.13E-05 | 0 (0 ,0) | NA | 2 | 2 | NP |
| Musa-Veloso, 2011[29] | long-chain n-3 fatty acid and sudden cardiac death | 3 | 35443 | 499 | 0.64(0.47,0.86) | 0.65 (0.54 ,0.79) | 9.80E-06 | 1.4 (0 ,73.3) | 0.43 | 2 | 2.62 | NP |
| Musa-Veloso, 2011[29] | long-chain n-3 fatty acid and fatal coronary events | 5 | 161708 | 1805 | 1.05(0.87,1.28) | 0.83 (0.68 ,1.03) | 8.46E-02 | 66 (0 ,84.9) | 0.24 | 2 | 0.42 | 0.06 |
| Pan, 2012[30] | dietary a-linolenic acid intake and risk of CVD death | 6 | 150438 | 3370 | 1.02(0.73,1.44) | 0.8 (0.65 ,0.98) | 3.46E-02 | 18.6 (0 ,67.8) | 0.77 | 1 | 0.34 | 0.30 |
| Pan, 2012[30] | a-linolenic acid biomarker concentration and risk of CVD death | 3 | 3668 | 1144 | 1.23(1,1.52) | 1.06 (0.64 ,1.75) | 8.23E-01 | 49.2 (0 ,84.3) | 0.38 | 0 | 0.98 | NP |
| Poggio, 2015[31] | sodium intake and cvd mortality | 11 | 199785 | 9346 | 1.05(0.96,1.15) | 1.16 (1.03 ,1.31) | 1.70E-02 | 68.4 (29.7 ,81.6) | 0.63 | 5 | 1.3 | 0.01 |
| Schwingshackl, 2014[32] | MUFA and all-cause mortality | 5 | 183956 | 20685 | 0.97(0.9,1.05) | 1 (0.93 ,1.08) | 9.32E-01 | 21.6 (0 ,71.3) | 0.67 | 0 | 0.84 | NP |
| Schwingshackl, 2014[32] | MUFA:SFA ratio and all-cause mortality | 10 | 246390 | 19410 | 0.98(0.91,1.06) | 0.9 (0.82 ,1) | 4.40E-02 | 58.5 (0 ,77.7) | 0.15 | 3 | 0.74 | 0.03 |
| Schwingshackl, 2014[32] | olive oil and all-cause mortality | 4 | 73665 | 3446 | 0.77(0.68,0.87) | 0.77 (0.7 ,0.85) | 4.86E-07 | 0 (0 ,67.9) | 0.86 | 2 | 3.34 | NP |
| Schwingshackl, 2014[32] | MUFA and cvd mortality | 8 | 255053 | 4967 | 0.97(0.89,1.06) | 0.96 (0.89 ,1.04) | 3.69E-01 | 7 (0 ,59.3) | 0.65 | 0 | 0.56 | NP |
| Schwingshackl, 2014[32] | MUFA:SFA and cvd mortality | 4 | 103788 | 2346 | 0.92(0.81,1.04) | 0.91 (0.83 ,0.99) | 3.34E-02 | 0 (0 ,67.9) | 0.38 | 0 | 0.76 | NP |
| Schwingshackl, 2014[32] | olive oil and cvd mortality | 5 | 98252 | 1260 | 0.61(0.46,0.81) | 0.7 (0.48 ,1.03) | 7.05E-02 | 71.4 (0 ,86.7) | 0.87 | 3 | 4.64 | NP |
| Wang, 2014[33] | fruits and vegetables and all-cause mortality | 7 | 553698 | 42219 | 0.99(0.98,0.99) | 0.95 (0.92 ,0.98) | 6.64E-04 | 82.4 (60.6 ,89.8) | 0.01 | 5 | 0.49 | <0.01 |
| Wang, 2014[33] | fruits and all-cause mortality | 7 | 660186 | 40192 | 0.99(0.98,1) | 0.94 (0.9 ,0.98) | 2.07E-03 | 77.4 (42.5 ,87.5) | 0.02 | 4 | 0.48 | <0.01 |
| Wang, 2014[33] | vegetables and all-cause mortality | 7 | 660186 | 40192 | 0.97(0.96,0.98) | 0.95 (0.92 ,0.99) | 6.40E-03 | 86.1 (71.7 ,91.5) | 0.36 | 4 | 1.48 | 0.04 |
| Wang, 2014[33] | fruits and vegetables and cardiovascular mortality | 4 | 469551 | 6893 | 0.98(0.96,0.99) | 0.96 (0.92 ,0.99) | 1.65E-02 | 42.4 (0 ,79.8) | 0.08 | 2 | 0.3 | 0.03 |
| Wang, 2014[33] | fruits and cardiovascular mortality | 6 | 677674 | 9744 | 1(0.97,1.02) | 0.95 (0.91 ,1) | 3.41E-02 | 70.7 (0.5 ,85.5) | 0.40 | 3 | 0.3 | <0.01 |
| Wang, 2014[33] | vegetables and cardiovascular mortality | 6 | 677674 | 9744 | 0.93(0.91,0.96) | 0.96 (0.93 ,0.99) | 1.26E-02 | 62.7 (0 ,82.6) | 0.77 | 3 | 1.91 | 0.39 |
| Chen, 2016[34] | long-chain n-3 polyunsaturated x all cause mortality | 6 | 361273 | 27621 | 0.94(0.86,1.03) | 0.9 (0.83 ,0.97) | 9.56E-03 | 69 (0 ,84.9) | 0.59 | 2 | 3.42 | NP |
| Chen, 2016[34] | EPA x all cause mortality | 3 | 6410 | 3205 | 0.83(0.71,0.98) | 0.74 (0.6 ,0.9) | 3.11E-03 | 55.1 (0 ,85.6) | 0.37 | 2 | 1.88 | 1.00 |
| Chen, 2016[34] | DHA x all cause mortality | 3 | 6410 | 3205 | 0.8(0.67,0.94) | 0.78 (0.64 ,0.93) | 6.72E-03 | 38.3 (0 ,82) | 0.68 | 2 | 2.32 | NP |
| Cheng, 2015[35] | Long chain n-3 PUFA intake assessment and stroke mortality | 7 | 419938 | 4964 | 0.91(0.74,1.12) | 0.84 (0.73 ,0.97) | 1.79E-02 | 31.3 (0 ,70.3) | 0.08 | 2 | 1.94 | 0.96 |
| Cheng, 2016[36] | dietary saturated fat and stroke mortality | 3 | 74060 | 1428 | 0.67(0.49,0.92) | 0.71 (0.56 ,0.92) | 8.16E-03 | 0 (0 ,72.9) | 0.95 | 1 | 2.53 | NP |
| De Souza, 2015[37] | saturated fat intake and all-cause mortality | 5 | 99906 | 14305 | 0.95(0.89,1.01) | 1.04 (0.91 ,1.19) | 5.92E-01 | 47.9 (0 ,79.2) | 0.21 | 1 | 1.27 | NP |
| De Souza, 2015[37] | saturated fat intake and chd mortality | 11 | 103548 | 3295 | 1.1(0.96,1.26) | 1.15 (0.99 ,1.33) | 7.52E-02 | 59.3 (0.3 ,77.5) | 0.13 | 3 | 1.65 | 0.22 |
| De Souza, 2015[37] | saturated fat intake and cvd mortality | 3 | 90501 | 2094 | 1.02(0.91,1.14) | 0.9 (0.7 ,1.17) | 4.33E-01 | 39.8 (0 ,82.3) | 0.04 | 0 | 0.17 | NP |
| De Souza, 2015[37] | total trans fat and all-cause mortality | 2 | 20346 | 2140 | 1.24(1.04,1.47) | 1.42 (1.04 ,1.94) | 2.69E-02 | 69.9 (0 ,0) | NA | 2 | 1.65 | 1.00 |
| De Souza, 2015[37] | total trans fat and CHD mortality | 7 | 107610 | 3628 | 1.15(0.92,1.43) | 1.22 (1.07 ,1.38) | 2.17E-03 | 0 (0 ,58.5) | 0.38 | 1 | 2.37 | NP |
| De Souza, 2015[37] | industrial trans fat and CHD mortality | 2 | 93394 | 3018 | 1.16(1,1.34) | 1.18 (1.04 ,1.34) | 9.24E-03 | 0 (0 ,0) | NA | 1 | 1.51 | NP |
| De Souza, 2015[37] | ruminant trans fat and CHD mortality | 2 | 93394 | 3018 | 1.23(1.03,1.46) | 1.03 (0.7 ,1.51) | 8.87E-01 | 80.5 (0 ,0) | NA | 1 | 1.8 | NP |
| Narain, 2016[38] | sugar-sweetened beverage and all-cause mortality | 3 | 148800 | 47432 | 1.02(0.92,1.13) | 1.03 (0.91 ,1.18) | 6.19E-01 | 74.5 (0 ,90.3) | 0.97 | 1 | 0.57 | 0.47 |
| Narain, 2016[38] | artificially sweetened beverage intake and all-cause mortality | 2 | 96216 | 37403 | 1.18(1.07,1.3) | 1.09 (0.92 ,1.3) | 3.15E-01 | 72.7 (0 ,0) | NA | 1 | 2 | NP |
| *Statin* |  |  |  |  |  |  |  |  |  |  |  |  |
| Bukkapatnam, 2010[39] | statin and all-cause mortality | 3 | 11384 | 79 | 0.98(0.83,1.17) | 0.9 (0.6 ,1.35) | 6.07E-01 | 54.1 (0 ,85.4) | 0.89 | 1 | 1.1 | NP |
| Kizer, 2010[40] | statin and all-cause mortality | 11 | 95813 | 6820 | 0.86(0.8,0.93) | 0.9 (0.84 ,0.96) | 1.89E-03 | 30 (0 ,64.7) | 0.88 | 2 | 4.32 | NP |
| Kostis, 2012[41] | statin and all-cause mortality in men | 6 | 42647 | 3995 | 0.87(0.79,0.95) | 0.92 (0.85 ,1.01) | 7.97E-02 | 22 (0 ,69) | 0.73 | 1 | 2.21 | NP |
| Kostis, 2012[41] | statin and all-cause mortality in women | 6 | 26287 | 1496 | 0.94(0.79,1.13) | 0.87 (0.78 ,0.97) | 1.36E-02 | 3.2 (0 ,62.2) | 0.79 | 1 | 0.49 | 0.40 |
| Lv, 2014[42] | statin and all-cause mortality | 3 | 37436 | 6011 | 0.95(0.91,0.98) | 0.94 (0.9 ,0.97) | 1.21E-03 | 11.6 (0 ,75.9) | 0.24 | 2 | 0.63 | 0.11 |
| Lv, 2014[42] | statin and cvd mortality | 3 | 37436 | 4720 | 0.92(0.87,0.97) | 0.91 (0.87 ,0.96) | 3.16E-04 | 0 (0 ,72.9) | 0.50 | 2 | 1.04 | 0.28 |
| Ray, 2010[43] | statin and all-cause mortality | 11 | 65229 | 2833 | 1(0.88,1.13) | 0.92 (0.83 ,1.01) | 6.52E-02 | 21.5 (0 ,61.1) | 0.47 | 1 | 0.55 | 0.43 |
| Savarese, 2013[44] | statin and all-cause mortality | 7 | 31272 | 2511 | 1.01(0.89,1.15) | 0.94 (0.86 ,1.04) | 2.39E-01 | 0 (0 ,58.5) | 0.24 | 0 | 0.36 | NP |
| Savarese, 2013[44] | statin and cvd mortality | 5 | 19103 | 191 | 1.03(0.7,1.51) | 0.91 (0.69 ,1.2) | 4.95E-01 | 0 (0 ,64.1) | 0.59 | 0 | 0.26 | NP |
| Taylor, 2011[45] | statin and all-cause mortality | 8 | 28161 | 802 | 0.78(0.61,1.01) | 0.84 (0.72 ,0.99) | 3.34E-02 | 13.5 (0 ,61.9) | 0.76 | 0 | 2.13 | NP |
| Taylor, 2011[45] | statin and chd mortality | 7 | 17619 | 186 | 0.73(0.48,1.1) | 0.8 (0.6 ,1.06) | 1.19E-01 | 0.6 (0 ,58.7) | 0.81 | 0 | 1.01 | NP |
| Taylor, 2011[45] | statin and cvd mortality | 2 | 7459 | 131 | 0.68(0.48,0.98) | 0.7 (0.49 ,0.98) | 4.03E-02 | 0 (0 ,0) | NA | 1 | 0.74 | 1.00 |
| Tonelli, 2011[46] | low-dose statin and all-cause mortality | 13 | 48307 | 2059 | 0.99(0.89,1.09) | 0.9 (0.78 ,1.03) | 1.32E-01 | 24 (0 ,60.2) | 0.73 | 2 | 0.66 | 0.14 |
| Tonelli, 2011[46] | high-dose statin and all-cause mortality | 6 | 29997 | 878 | 0.8(0.67,0.96) | 0.84 (0.74 ,0.96) | 9.72E-03 | 0 (0 ,61) | 0.16 | 1 | 1.66 | NP |
| Chou, 2016[47] | statin and all-cause mortality | 14 | 129731 | 2351 | 0.93(0.81,1.08) | 0.86 (0.8 ,0.93) | 2.07E-04 | 0 (0 ,47.4) | 0.49 | 3 | 1.13 | 0.10 |
| Chou, 2016[47] | statin and cvd mortality | 10 | 110847 | 947 | 0.9(0.72,1.11) | 0.69 (0.54 ,0.88) | 2.66E-03 | 53.6 (0 ,75.6) | 0.25 | 3 | 0.87 | 0.05 |
| Preiss, 2015[48] | statin and heart failure death | 5 | 47200 | 33 | 0.75(0.26,2.16) | 0.75 (0.38 ,1.49) | 4.12E-01 | 0 (0 ,64.1) | 0.85 | 0 | 0.35 | NP |
| Teng, 2015[49] | statin and all-cause mortality | 7 | 23357 | 2710 | 1.01(0.91,1.13) | 0.96 (0.88 ,1.04) | 2.97E-01 | 0 (0 ,58.5) | 0.12 | 0 | 0.36 | NP |
| Teng, 2015[49] | statin and stroke mortality | 2 | 6938 | 114 | 1.04(0.69,1.55) | 0.74 (0.22 ,2.49) | 6.26E-01 | 42.5 (0 ,0) | NA | 0 | 0.11 | NP |
| Teng, 2015[49] | statin and myocardical infarction mortality | 2 | 6938 | 68 | 0.81(0.58,1.13) | 0.42 (0.09 ,2.01) | 2.78E-01 | 78.2 (0 ,0) | NA | 1 | 0.21 | 0.20 |

Abbreviations: CI, confidence interval; NA, not applicable, because the numbers of studies are less than three; NP, not pertinent, because the expected number of significant studies is larger than the observed; RR, relative risk; O, Observed number of statistically significant studies; E, Expected number of statistically significant studies

**^**^**Random effects refer to summary risk ratio (95% CI) using the random-effects model.

^#^ P*-*value of the summary random effects estimate expressed in scientific notation.

**^†^** P-value from the Egger’s regression asymmetry test.

**^‡^** Expected number of statistically significant studies using the point estimate of the largest study (smallest SE) as the plausible effect size.

**^⁑^** *P*-value of the excess statistical significance test.

**REFERENCES**

1. Kelly P, Kahlmeier S, Gotschi T, Orsini N, Richards J, Roberts N, et al. Systematic review and meta-analysis of reduction in all-cause mortality from walking and cycling and shape of dose response relationship. Int J Behav Nutr Phys Act. 2014;11:132. doi: 10.1186/s12966-014-0132-x.

2. Samitz G, Egger M, Zwahlen M. Domains of physical activity and all-cause mortality: systematic review and dose-response meta-analysis of cohort studies. Int J Epidemiol. 2011;40(5):1382-400. doi: 10.1093/ije/dyr112.

3. Woodcock J, Franco OH, Orsini N, Roberts I. Non-vigorous physical activity and all-cause mortality: systematic review and meta-analysis of cohort studies. Int J Epidemiol. 2011;40(1):121-38. doi: 10.1093/ije/dyq104.

4. Hupin D, Roche F, Gremeaux V, Chatard JC, Oriol M, Gaspoz JM, et al. Even a low-dose of moderate-to-vigorous physical activity reduces mortality by 22% in adults aged >/=60 years: a systematic review and meta-analysis. Br J Sports Med. 2015;49(19):1262-7. doi: 10.1136/bjsports-2014-094306.

5. Biswas A, Oh PI, Faulkner GE, Bajaj RR, Silver MA, Mitchell MS, et al. Sedentary time and its association with risk for disease incidence, mortality, and hospitalization in adults: a systematic review and meta-analysis. Ann Intern Med. 2015;162(2):123-32. doi: 10.7326/M14-1651.

6. Chau JY, Grunseit AC, Chey T, Stamatakis E, Brown WJ, Matthews CE, et al. Daily sitting time and all-cause mortality: a meta-analysis. PLoS One. 2013;8(11):e80000. doi: 10.1371/journal.pone.0080000.

7. Grontved A, Hu FB. Television viewing and risk of type 2 diabetes, cardiovascular disease, and all-cause mortality: a meta-analysis. JAMA. 2011;305(23):2448-55. doi: 10.1001/jama.2011.812.

8. Wilmot EG, Edwardson CL, Achana FA, Davies MJ, Gorely T, Gray LJ, et al. Sedentary time in adults and the association with diabetes, cardiovascular disease and death: systematic review and meta-analysis. Diabetologia. 2012;55(11):2895-905. doi: 10.1007/s00125-012-2677-z.

9. Ford ES, Caspersen CJ. Sedentary behaviour and cardiovascular disease: a review of prospective studies. Int J Epidemiol. 2012;41(5):1338-53. doi: 10.1093/ije/dys078.

10. Pandey A, Salahuddin U, Garg S, Ayers C, Kulinski J, Anand V, et al. Continuous Dose-Response Association Between Sedentary Time and Risk for Cardiovascular Disease: A Meta-analysis. JAMA Cardiol. 2016;1(5):575-83.ndoi: 10.1001/jamacardio.2016.1567.

11. Sun JW, Zhao LG, Yang Y, Ma X, Wang YY, Xiang YB. Association Between Television Viewing Time and All-Cause Mortality: A Meta-Analysis of Cohort Studies. Am J Epidemiol. 2015;182(11):908-16. doi: 10.1093/aje/kwv164.

12. Costanzo S, Di Castelnuovo A, Donati MB, Iacoviello L, de Gaetano G. Wine, beer or spirit drinking in relation to fatal and non-fatal cardiovascular events: a meta-analysis. Eur J Epidemiol. 2011;26(11):833-50. doi: 10.1007/s10654-011-9631-0.

13. Jayasekara H, English DR, Room R, MacInnis RJ. Alcohol consumption over time and risk of death: a systematic review and meta-analysis. Am J Epidemiol. 2014;179(9):1049-59. doi: 10.1093/aje/kwu028.

14. Roerecke M, Rehm J. Ischemic heart disease mortality and morbidity rates in former drinkers: a meta-analysis. American journal of epidemiology. 2011;173(3):245-58. doi: 10.1093/aje/kwq364.

15. Roerecke M, Rehm J. Alcohol consumption, drinking patterns, and ischemic heart disease: a narrative review of meta-analyses and a systematic review and meta-analysis of the impact of heavy drinking occasions on risk for moderate drinkers. BMC Med. 2014;12:182. doi: 10.1186/s12916-014-0182-6.

16. Ronksley PE, Brien SE, Turner BJ, Mukamal KJ, Ghali WA. Association of alcohol consumption with selected cardiovascular disease outcomes: a systematic review and meta-analysis. BMJ. 2011;342:d671. doi: 10.1136/bmj.d671.

17. Park JE, Choi TY, Ryu Y, Cho SI. The relationship between mild alcohol consumption and mortality in Koreans: a systematic review and meta-analysis. BMC Public Health. 2015;15:918. doi: 10.1186/s12889-015-2263-7.

18. Stockwell T, Zhao J, Panwar S, Roemer A, Naimi T, Chikritzhs T. Do "Moderate" Drinkers Have Reduced Mortality Risk? A Systematic Review and Meta-Analysis of Alcohol Consumption and All-Cause Mortality. J Stud Alcohol Drugs. 2016;77(2):185-98.

19. Zheng YL, Lian F, Shi Q, Zhang C, Chen YW, Zhou YH, et al. Alcohol intake and associated risk of major cardiovascular outcomes in women compared with men: a systematic review and meta-analysis of prospective observational studies. BMC Public Health. 2015;15:773. doi: 10.1186/s12889-015-2081-y.

20. Roerecke M, Rehm J. Irregular heavy drinking occasions and risk of ischemic heart disease: a systematic review and meta-analysis. Am J Epidemiol. 2010;171(6):633-44. doi: 10.1093/aje/kwp451.

21. Roerecke M, Rehm J. Chronic heavy drinking and ischaemic heart disease: a systematic review and meta-analysis. Open Heart. 2014;1(1):e000135. doi: 10.1136/openhrt-2014-000135.

22. Gellert C, Schottker B, Brenner H. Smoking and all-cause mortality in older people: systematic review and meta-analysis. Arch Intern Med. 2012;172(11):837-44. doi: 10.1001/archinternmed.2012.1397.

23. Lv X, Sun J, Bi Y, Xu M, Lu J, Zhao L, et al. Risk of all-cause mortality and cardiovascular disease associated with secondhand smoke exposure: a systematic review and meta-analysis. Int J Cardiol. 2015;199:106-15. doi: 10.1016/j.ijcard.2015.07.011.

24. Sinha DN, Suliankatchi RA, Gupta PC, Thamarangsi T, Agarwal N, Parascandola M, et al. Global burden of all-cause and cause-specific mortality due to smokeless tobacco use: systematic review and meta-analysis. Tob Control. 2018;27(1):35-42. doi: 10.1136/tobaccocontrol-2016-053302.

25. Farvid MS, Ding M, Pan A, Sun Q, Chiuve SE, Steffen LM, et al. Dietary linoleic acid and risk of coronary heart disease: a systematic review and meta-analysis of prospective cohort studies. Circulation. 2014;130(18):1568-78. doi: 10.1161/CIRCULATIONAHA.114.010236.

26. Graudal N, Jurgens G, Baslund B, Alderman MH. Compared with usual sodium intake, low- and excessive-sodium diets are associated with increased mortality: a meta-analysis. Am J Hypertens. 2014;27(9):1129-37. doi: 10.1093/ajh/hpu028.

27. Hu D, Huang J, Wang Y, Zhang D, Qu Y. Fruits and vegetables consumption and risk of stroke: a meta-analysis of prospective cohort studies. Stroke. 2014;45(6):1613-9. doi: 10.1161/STROKEAHA.114.004836.

28. Li XY, Cai XL, Bian PD, Hu LR. High salt intake and stroke: meta-analysis of the epidemiologic evidence. CNS Neurosci Ther. 2012;18(8):691-701. doi: 10.1111/j.1755-5949.2012.00355.x.

29. Musa-Veloso K, Binns MA, Kocenas A, Chung C, Rice H, Oppedal-Olsen H, et al. Impact of low v. moderate intakes of long-chain n-3 fatty acids on risk of coronary heart disease. Br J Nutr. 2011;106(8):1129-41. doi: 10.1017/S0007114511001644.

30. Pan A, Chen M, Chowdhury R, Wu JH, Sun Q, Campos H, et al. alpha-Linolenic acid and risk of cardiovascular disease: a systematic review and meta-analysis. Am J Clin Nutr. 2012;96(6):1262-73. doi: 10.3945/ajcn.112.044040.

31. Poggio R, Gutierrez L, Matta MG, Elorriaga N, Irazola V, Rubinstein A. Daily sodium consumption and CVD mortality in the general population: systematic review and meta-analysis of prospective studies. Public Health Nutr. 2015;18(4):695-704. doi: 10.1017/S1368980014000949.

32. Schwingshackl L, Hoffmann G. Monounsaturated fatty acids, olive oil and health status: a systematic review and meta-analysis of cohort studies. Lipids Health Dis. 2014;13:154. doi: 10.1186/1476-511X-13-154.

33. Wang X, Ouyang Y, Liu J, Zhu M, Zhao G, Bao W, et al. Fruit and vegetable consumption and mortality from all causes, cardiovascular disease, and cancer: systematic review and dose-response meta-analysis of prospective cohort studies. BMJ. 2014;349:g4490. doi: 10.1136/bmj.g4490.

34. Chen GC, Yang J, Eggersdorfer M, Zhang W, Qin LQ. N-3 long-chain polyunsaturated fatty acids and risk of all-cause mortality among general populations: a meta-analysis. Sci Rep. 2016;6:28165. doi: 10.1038/srep28165.

35. Cheng P, Huang W, Bai S, Wu Y, Yu J, Zhu X, et al. BMI Affects the Relationship between Long Chain N-3 Polyunsaturated Fatty Acid Intake and Stroke Risk: a Meta-Analysis. Sci Rep. 2015;5:14161.

36. Cheng P, Wang J, Shao W, Liu M, Zhang H. Can dietary saturated fat be beneficial in prevention of stroke risk? A meta-analysis. Neurol Sci. 2016;37(7):1089-98. doi: 10.1007/s10072-016-2548-3.

37. de Souza RJ, Mente A, Maroleanu A, Cozma AI, Ha V, Kishibe T, et al. Intake of saturated and trans unsaturated fatty acids and risk of all cause mortality, cardiovascular disease, and type 2 diabetes: systematic review and meta-analysis of observational studies. BMJ. 2015;351:h3978. Epub 2015/08/14. doi: 10.1136/bmj.h3978.

38. Narain A, Kwok CS, Mamas MA. Soft drinks and sweetened beverages and the risk of cardiovascular disease and mortality: a systematic review and meta-analysis. Int J Clin Pract. 2016;70(10):791-805. doi: 10.1111/ijcp.12841.

39. Bukkapatnam RN, Gabler NB, Lewis WR. Statins for primary prevention of cardiovascular mortality in women: a systematic review and meta-analysis. Prev Cardiol. 2010;13(2):84-90. doi: 10.1111/j.1751-7141.2009.00059.x.

40. Kizer JR, Madias C, Wilner B, Vaughan CJ, Mushlin AI, Trushin P, et al. Relation of different measures of low-density lipoprotein cholesterol to risk of coronary artery disease and death in a meta-regression analysis of large-scale trials of statin therapy. Am J Cardiol. 2010;105(9):1289-96. doi: 10.1016/j.amjcard.2009.12.051.

41. Kostis WJ, Cheng JQ, Dobrzynski JM, Cabrera J, Kostis JB. Meta-analysis of statin effects in women versus men. J Am Coll Cardiol. 2012;59(6):572-82. doi: 10.1016/j.jacc.2011.09.067.

42. Lv HL, Jin DM, Liu M, Liu YM, Wang JF, Geng DF. Long-term efficacy and safety of statin treatment beyond six years: a meta-analysis of randomized controlled trials with extended follow-up. Pharmacol Res. 2014;81:64-73. doi: 10.1016/j.phrs.2014.02.006.

43. Ray KK, Seshasai SR, Erqou S, Sever P, Jukema JW, Ford I, et al. Statins and all-cause mortality in high-risk primary prevention: a meta-analysis of 11 randomized controlled trials involving 65,229 participants. Arch Intern Med. 2010;170(12):1024-31. doi: 10.1001/archinternmed.2010.182.

44. Savarese G, Gotto AM, Jr., Paolillo S, D'Amore C, Losco T, Musella F, et al. Benefits of statins in elderly subjects without established cardiovascular disease: a meta-analysis. J Am Coll Cardiol. 2013;62(22):2090-9. doi: 10.1016/j.jacc.2013.07.069.

45. Taylor F, Ward K, Moore TH, Burke M, Davey Smith G, Casas JP, et al. Statins for the primary prevention of cardiovascular disease. Cochrane Database Syst Rev. 2011;(1):CD004816. doi: 10.1002/14651858.CD004816.pub4.

46. Tonelli M, Lloyd A, Clement F, Conly J, Husereau D, Hemmelgarn B, et al. Efficacy of statins for primary prevention in people at low cardiovascular risk: a meta-analysis. CMAJ. 2011;183(16):E1189-202. doi: 10.1503/cmaj.101280.

47. Chou R, Dana T, Blazina I, Daeges M, Jeanne TL. Statins for Prevention of Cardiovascular Disease in Adults: Evidence Report and Systematic Review for the US Preventive Services Task Force. JAMA. 2016;316(19):2008-24. doi: 10.1001/jama.2015.15629.

48. Preiss D, Campbell RT, Murray HM, Ford I, Packard CJ, Sattar N, et al. The effect of statin therapy on heart failure events: a collaborative meta-analysis of unpublished data from major randomized trials. Eur Heart J. 2015;36(24):1536-46. doi: 10.1093/eurheartj/ehv072.

49. Teng M, Lin L, Zhao YJ, Khoo AL, Davis BR, Yong QW, et al. Statins for Primary Prevention of Cardiovascular Disease in Elderly Patients: Systematic Review and Meta-Analysis. Drugs Aging. 2015;32(8):649-61. doi: 10.1007/s40266-015-0290-9.
