# Supplementary material for: Reporting bias in the literature on the associations of health-related behaviors and statins with cardiovascular disease and all-cause mortality
Source: PLoS Biol. 2018 Jun 18;16(6):e2005761. doi: 10.1371/journal.pbio.2005761 (PMC6023226; doi:10.1371/journal.pbio.2005761)
Supplement: S5 Table — Relative and absolute frequencies of meta-analyses with nominally statistically significant results, small-study effects, and excess significance, by research area. (DOC) [file pbio.2005761.s007.doc]

| **S5 Table:** Sensitivity analysis excluding individual studies with less than 200 deaths. Relative and absolute frequencies of meta-analyses with nominally statistical significant results, small-study effects, and excess significance, by research area | | | | | | | | | | | | | | | |
| --- | --- | --- | --- | --- | --- | --- | --- | --- | --- | --- | --- | --- | --- | --- | --- |
| **Research area** | **Total number of meta-analysis** |  | **Meta-analysis with P<0.05 (%)** | |  | **Small-study effects** | | |  | **Excess significance** | | | | | |
|  |  |  |  |  |  |  |  |  |  |  | **O>E** | | | | |
|  | **N** |  | **n** | **%** |  | **N*** | **n** | **%** |  | **N**** | **n** | **%** |  | **n with P<0.05** | **%** |
| *Physical Activity* | 12 |  | 12 | 100 |  | 12 | 3 | 25 |  | 12 | 8 | 67 |  | 6 | 50 |
| *Sedentary behaviour* | 11 |  | 11 | 100 |  | 11 | 0 | 0 |  | 11 | 7 | 64 |  | 1 | 9 |
| *Alcohol* | 24 |  | 9 | 38 |  | 13 | 2 | 15 |  | 11 | 3 | 27 |  | 1 | 9 |
| *Smoking* | 7 |  | 7 | 100 |  | 7 | 3 | 43 |  | 7 | 4 | 57 |  | 0 | 0 |
| *Diet* | 36 |  | 24 | 67 |  | 27 | 4 | 15 |  | 27 | 15 | 56 |  | 9 | 33 |
| *Statin* | 14 |  | 5 | 36 |  | 8 | 1 | 13 |  | 8 | 4 | 50 |  | 0 | 0 |
| Overall | 104 |  | 68 | 65 |  | 78 | 13 | 17 |  | 76 | 41 | 54 |  | 17 | 22 |

1. Kelly P, Kahlmeier S, Gotschi T, Orsini N, Richards J, Roberts N, et al. Systematic review and meta-analysis of reduction in all-cause mortality from walking and cycling and shape of dose response relationship. Int J Behav Nutr Phys Act. 2014;11:132. doi: 10.1186/s12966-014-0132-x. PubMed PMID: 25344355; PubMed Central PMCID: PMCPMC4262114.

2. Samitz G, Egger M, Zwahlen M. Domains of physical activity and all-cause mortality: systematic review and dose-response meta-analysis of cohort studies. Int J Epidemiol. 2011;40(5):1382-400. doi: 10.1093/ije/dyr112. PubMed PMID: 22039197.

3. Woodcock J, Franco OH, Orsini N, Roberts I. Non-vigorous physical activity and all-cause mortality: systematic review and meta-analysis of cohort studies. Int J Epidemiol. 2011;40(1):121-38. doi: 10.1093/ije/dyq104. PubMed PMID: 20630992.

4. Hupin D, Roche F, Gremeaux V, Chatard JC, Oriol M, Gaspoz JM, et al. Even a low-dose of moderate-to-vigorous physical activity reduces mortality by 22% in adults aged >/=60 years: a systematic review and meta-analysis. Br J Sports Med. 2015;49(19):1262-7. doi: 10.1136/bjsports-2014-094306. PubMed PMID: 26238869.

5. Biswas A, Oh PI, Faulkner GE, Bajaj RR, Silver MA, Mitchell MS, et al. Sedentary time and its association with risk for disease incidence, mortality, and hospitalization in adults: a systematic review and meta-analysis. Ann Intern Med. 2015;162(2):123-32. doi: 10.7326/M14-1651. PubMed PMID: 25599350.

6. Chau JY, Grunseit AC, Chey T, Stamatakis E, Brown WJ, Matthews CE, et al. Daily sitting time and all-cause mortality: a meta-analysis. PLoS One. 2013;8(11):e80000. doi: 10.1371/journal.pone.0080000. PubMed PMID: 24236168; PubMed Central PMCID: PMCPMC3827429.

7. Grontved A, Hu FB. Television viewing and risk of type 2 diabetes, cardiovascular disease, and all-cause mortality: a meta-analysis. JAMA. 2011;305(23):2448-55. doi: 10.1001/jama.2011.812. PubMed PMID: 21673296; PubMed Central PMCID: PMCPMC4324728.

8. Wilmot EG, Edwardson CL, Achana FA, Davies MJ, Gorely T, Gray LJ, et al. Sedentary time in adults and the association with diabetes, cardiovascular disease and death: systematic review and meta-analysis. Diabetologia. 2012;55(11):2895-905. doi: 10.1007/s00125-012-2677-z. PubMed PMID: 22890825.

9. Ford ES, Caspersen CJ. Sedentary behaviour and cardiovascular disease: a review of prospective studies. Int J Epidemiol. 2012;41(5):1338-53. doi: 10.1093/ije/dys078. PubMed PMID: 22634869; PubMed Central PMCID: PMCPMC4582407.

10. Pandey A, Salahuddin U, Garg S, Ayers C, Kulinski J, Anand V, et al. Continuous Dose-Response Association Between Sedentary Time and Risk for Cardiovascular Disease: A Meta-analysis. JAMA cardiology. 2016;1(5):575-83. Epub 2016/07/20. doi: 10.1001/jamacardio.2016.1567. PubMed PMID: 27434872.

11. Sun JW, Zhao LG, Yang Y, Ma X, Wang YY, Xiang YB. Association Between Television Viewing Time and All-Cause Mortality: A Meta-Analysis of Cohort Studies. American journal of epidemiology. 2015;182(11):908-16. Epub 2015/11/17. doi: 10.1093/aje/kwv164. PubMed PMID: 26568572.

12. Costanzo S, Di Castelnuovo A, Donati MB, Iacoviello L, de Gaetano G. Wine, beer or spirit drinking in relation to fatal and non-fatal cardiovascular events: a meta-analysis. Eur J Epidemiol. 2011;26(11):833-50. doi: 10.1007/s10654-011-9631-0. PubMed PMID: 22076059.

13. Jayasekara H, English DR, Room R, MacInnis RJ. Alcohol consumption over time and risk of death: a systematic review and meta-analysis. American journal of epidemiology. 2014;179(9):1049-59. doi: 10.1093/aje/kwu028. PubMed PMID: 24670372.

14. Roerecke M, Rehm J. Ischemic heart disease mortality and morbidity rates in former drinkers: a meta-analysis. American journal of epidemiology. 2011;173(3):245-58. doi: 10.1093/aje/kwq364. PubMed PMID: 21156750; PubMed Central PMCID: PMCPMC3105267.

15. Roerecke M, Rehm J. Alcohol consumption, drinking patterns, and ischemic heart disease: a narrative review of meta-analyses and a systematic review and meta-analysis of the impact of heavy drinking occasions on risk for moderate drinkers. BMC Med. 2014;12:182. doi: 10.1186/s12916-014-0182-6. PubMed PMID: 25567363; PubMed Central PMCID: PMCPMC4203905.

16. Ronksley PE, Brien SE, Turner BJ, Mukamal KJ, Ghali WA. Association of alcohol consumption with selected cardiovascular disease outcomes: a systematic review and meta-analysis. BMJ. 2011;342:d671. doi: 10.1136/bmj.d671. PubMed PMID: 21343207; PubMed Central PMCID: PMCPMC3043109.

17. Park JE, Choi TY, Ryu Y, Cho SI. The relationship between mild alcohol consumption and mortality in Koreans: a systematic review and meta-analysis. BMC public health. 2015;15:918. Epub 2015/09/20. doi: 10.1186/s12889-015-2263-7. PubMed PMID: 26385795; PubMed Central PMCID: PMCPMC4575439.

18. Stockwell T, Zhao J, Panwar S, Roemer A, Naimi T, Chikritzhs T. Do "Moderate" Drinkers Have Reduced Mortality Risk? A Systematic Review and Meta-Analysis of Alcohol Consumption and All-Cause Mortality. Journal of studies on alcohol and drugs. 2016;77(2):185-98. Epub 2016/03/22. PubMed PMID: 26997174; PubMed Central PMCID: PMCPMC4803651.

19. Zheng YL, Lian F, Shi Q, Zhang C, Chen YW, Zhou YH, et al. Alcohol intake and associated risk of major cardiovascular outcomes in women compared with men: a systematic review and meta-analysis of prospective observational studies. BMC public health. 2015;15:773. doi: 10.1186/s12889-015-2081-y. PubMed PMID: 26264040; PubMed Central PMCID: PMCPMC4533962.

20. Roerecke M, Rehm J. Irregular heavy drinking occasions and risk of ischemic heart disease: a systematic review and meta-analysis. American journal of epidemiology. 2010;171(6):633-44. doi: 10.1093/aje/kwp451. PubMed PMID: 20142394.

21. Roerecke M, Rehm J. Chronic heavy drinking and ischaemic heart disease: a systematic review and meta-analysis. Open Heart. 2014;1(1):e000135. doi: 10.1136/openhrt-2014-000135. PubMed PMID: 25332827; PubMed Central PMCID: PMCPMC4189294.

22. Gellert C, Schottker B, Brenner H. Smoking and all-cause mortality in older people: systematic review and meta-analysis. Arch Intern Med. 2012;172(11):837-44. doi: 10.1001/archinternmed.2012.1397. PubMed PMID: 22688992.

23. Lv X, Sun J, Bi Y, Xu M, Lu J, Zhao L, et al. Risk of all-cause mortality and cardiovascular disease associated with secondhand smoke exposure: a systematic review and meta-analysis. Int J Cardiol. 2015;199:106-15. doi: 10.1016/j.ijcard.2015.07.011. PubMed PMID: 26188829.

24. Sinha DN, Suliankatchi RA, Gupta PC, Thamarangsi T, Agarwal N, Parascandola M, et al. Global burden of all-cause and cause-specific mortality due to smokeless tobacco use: systematic review and meta-analysis. Tobacco control. 2016. Epub 2016/12/03. doi: 10.1136/tobaccocontrol-2016-053302. PubMed PMID: 27903956.

25. Farvid MS, Ding M, Pan A, Sun Q, Chiuve SE, Steffen LM, et al. Dietary linoleic acid and risk of coronary heart disease: a systematic review and meta-analysis of prospective cohort studies. Circulation. 2014;130(18):1568-78. doi: 10.1161/CIRCULATIONAHA.114.010236. PubMed PMID: 25161045; PubMed Central PMCID: PMCPMC4334131.

26. Graudal N, Jurgens G, Baslund B, Alderman MH. Compared with usual sodium intake, low- and excessive-sodium diets are associated with increased mortality: a meta-analysis. Am J Hypertens. 2014;27(9):1129-37. doi: 10.1093/ajh/hpu028. PubMed PMID: 24651634.

27. Hu D, Huang J, Wang Y, Zhang D, Qu Y. Fruits and vegetables consumption and risk of stroke: a meta-analysis of prospective cohort studies. Stroke. 2014;45(6):1613-9. doi: 10.1161/STROKEAHA.114.004836. PubMed PMID: 24811336.

28. Li XY, Cai XL, Bian PD, Hu LR. High salt intake and stroke: meta-analysis of the epidemiologic evidence. CNS Neurosci Ther. 2012;18(8):691-701. doi: 10.1111/j.1755-5949.2012.00355.x. PubMed PMID: 22742770.

29. Musa-Veloso K, Binns MA, Kocenas A, Chung C, Rice H, Oppedal-Olsen H, et al. Impact of low v. moderate intakes of long-chain n-3 fatty acids on risk of coronary heart disease. Br J Nutr. 2011;106(8):1129-41. doi: 10.1017/S0007114511001644. PubMed PMID: 21736820.

30. Pan A, Chen M, Chowdhury R, Wu JH, Sun Q, Campos H, et al. alpha-Linolenic acid and risk of cardiovascular disease: a systematic review and meta-analysis. Am J Clin Nutr. 2012;96(6):1262-73. doi: 10.3945/ajcn.112.044040. PubMed PMID: 23076616; PubMed Central PMCID: PMCPMC3497923.

31. Poggio R, Gutierrez L, Matta MG, Elorriaga N, Irazola V, Rubinstein A. Daily sodium consumption and CVD mortality in the general population: systematic review and meta-analysis of prospective studies. Public Health Nutr. 2015;18(4):695-704. doi: 10.1017/S1368980014000949. PubMed PMID: 24848764.

32. Schwingshackl L, Hoffmann G. Monounsaturated fatty acids, olive oil and health status: a systematic review and meta-analysis of cohort studies. Lipids Health Dis. 2014;13:154. doi: 10.1186/1476-511X-13-154. PubMed PMID: 25274026; PubMed Central PMCID: PMCPMC4198773.

33. Wang X, Ouyang Y, Liu J, Zhu M, Zhao G, Bao W, et al. Fruit and vegetable consumption and mortality from all causes, cardiovascular disease, and cancer: systematic review and dose-response meta-analysis of prospective cohort studies. Bmj. 2014;349:g4490. Epub 2014/07/31. doi: 10.1136/bmj.g4490. PubMed PMID: 25073782; PubMed Central PMCID: PMCPMC4115152.

34. Chen GC, Yang J, Eggersdorfer M, Zhang W, Qin LQ. N-3 long-chain polyunsaturated fatty acids and risk of all-cause mortality among general populations: a meta-analysis. Scientific reports. 2016;6:28165. Epub 2016/06/17. doi: 10.1038/srep28165. PubMed PMID: 27306836; PubMed Central PMCID: PMCPMC4910132.

35. Cheng P, Huang W, Bai S, Wu Y, Yu J, Zhu X, et al. BMI Affects the Relationship between Long Chain N-3 Polyunsaturated Fatty Acid Intake and Stroke Risk: a Meta-Analysis. Scientific reports. 2015;5:14161. Epub 2015/09/16. doi: 10.1038/srep14161. PubMed PMID: 26369699; PubMed Central PMCID: PMCPMC4572932.

36. Cheng P, Wang J, Shao W, Liu M, Zhang H. Can dietary saturated fat be beneficial in prevention of stroke risk? A meta-analysis. Neurological sciences : official journal of the Italian Neurological Society and of the Italian Society of Clinical Neurophysiology. 2016;37(7):1089-98. Epub 2016/03/17. doi: 10.1007/s10072-016-2548-3. PubMed PMID: 26979840.

37. de Souza RJ, Mente A, Maroleanu A, Cozma AI, Ha V, Kishibe T, et al. Intake of saturated and trans unsaturated fatty acids and risk of all cause mortality, cardiovascular disease, and type 2 diabetes: systematic review and meta-analysis of observational studies. Bmj. 2015;351:h3978. Epub 2015/08/14. doi: 10.1136/bmj.h3978. PubMed PMID: 26268692; PubMed Central PMCID: PMCPMC4532752.

38. Narain A, Kwok CS, Mamas MA. Soft drinks and sweetened beverages and the risk of cardiovascular disease and mortality: a systematic review and meta-analysis. Int J Clin Pract. 2016;70(10):791-805. doi: 10.1111/ijcp.12841. PubMed PMID: 27456347.

39. Bukkapatnam RN, Gabler NB, Lewis WR. Statins for primary prevention of cardiovascular mortality in women: a systematic review and meta-analysis. Prev Cardiol. 2010;13(2):84-90. doi: 10.1111/j.1751-7141.2009.00059.x. PubMed PMID: 20377811.

40. Kizer JR, Madias C, Wilner B, Vaughan CJ, Mushlin AI, Trushin P, et al. Relation of different measures of low-density lipoprotein cholesterol to risk of coronary artery disease and death in a meta-regression analysis of large-scale trials of statin therapy. Am J Cardiol. 2010;105(9):1289-96. doi: 10.1016/j.amjcard.2009.12.051. PubMed PMID: 20403481; PubMed Central PMCID: PMCPMC2917836.

41. Kostis WJ, Cheng JQ, Dobrzynski JM, Cabrera J, Kostis JB. Meta-analysis of statin effects in women versus men. J Am Coll Cardiol. 2012;59(6):572-82. doi: 10.1016/j.jacc.2011.09.067. PubMed PMID: 22300691.

42. Lv HL, Jin DM, Liu M, Liu YM, Wang JF, Geng DF. Long-term efficacy and safety of statin treatment beyond six years: a meta-analysis of randomized controlled trials with extended follow-up. Pharmacol Res. 2014;81:64-73. doi: 10.1016/j.phrs.2014.02.006. PubMed PMID: 24602799.

43. Ray KK, Seshasai SR, Erqou S, Sever P, Jukema JW, Ford I, et al. Statins and all-cause mortality in high-risk primary prevention: a meta-analysis of 11 randomized controlled trials involving 65,229 participants. Arch Intern Med. 2010;170(12):1024-31. doi: 10.1001/archinternmed.2010.182. PubMed PMID: 20585067.

44. Savarese G, Gotto AM, Jr., Paolillo S, D'Amore C, Losco T, Musella F, et al. Benefits of statins in elderly subjects without established cardiovascular disease: a meta-analysis. J Am Coll Cardiol. 2013;62(22):2090-9. Epub 2013/08/21. doi: 10.1016/j.jacc.2013.07.069. PubMed PMID: 23954343.

45. Taylor F, Ward K, Moore TH, Burke M, Davey Smith G, Casas JP, et al. Statins for the primary prevention of cardiovascular disease. Cochrane Database Syst Rev. 2011;(1):CD004816. doi: 10.1002/14651858.CD004816.pub4. PubMed PMID: 21249663; PubMed Central PMCID: PMCPMC4164175.

46. Tonelli M, Lloyd A, Clement F, Conly J, Husereau D, Hemmelgarn B, et al. Efficacy of statins for primary prevention in people at low cardiovascular risk: a meta-analysis. CMAJ : Canadian Medical Association journal = journal de l'Association medicale canadienne. 2011;183(16):E1189-202. Epub 2011/10/13. doi: 10.1503/cmaj.101280. PubMed PMID: 21989464; PubMed Central PMCID: PMCPMC3216447.

47. Chou R, Dana T, Blazina I, Daeges M, Jeanne TL. Statins for Prevention of Cardiovascular Disease in Adults: Evidence Report and Systematic Review for the US Preventive Services Task Force. JAMA. 2016;316(19):2008-24. doi: 10.1001/jama.2015.15629. PubMed PMID: 27838722.

48. Preiss D, Campbell RT, Murray HM, Ford I, Packard CJ, Sattar N, et al. The effect of statin therapy on heart failure events: a collaborative meta-analysis of unpublished data from major randomized trials. Eur Heart J. 2015;36(24):1536-46. doi: 10.1093/eurheartj/ehv072. PubMed PMID: 25802390; PubMed Central PMCID: PMCPMC4769322.

49. Teng M, Lin L, Zhao YJ, Khoo AL, Davis BR, Yong QW, et al. Statins for Primary Prevention of Cardiovascular Disease in Elderly Patients: Systematic Review and Meta-Analysis. Drugs Aging. 2015;32(8):649-61. doi: 10.1007/s40266-015-0290-9. PubMed PMID: 26245770.
